# The empirical evidence underpinning the concept and practice of person-centred care for serious illness: a systematic review

**DOI:** 10.1136/bmjgh-2020-003330

**Published:** 2020-12-10

**Authors:** Alessandra Giusti, Kennedy Nkhoma, Ruwayda Petrus, Inge Petersen, Liz Gwyther, Lindsay Farrant, Sridhar Venkatapuram, Richard Harding

**Affiliations:** 1 Cicely Saunders Institute of Palliative Care, Policy and Rehabilitation, King's College London, London, UK; 2 King's Global Health Institute, King's College London, London, UK; 3 School of Applied Human Sciences, University of KwaZulu-Natal College of Humanities, Durban, South Africa; 4 School of Public Health and Family Medicine, University of Cape Town Faculty of Health Sciences, Cape Town, Western Cape, South Africa

**Keywords:** systematic review, health services research, health systems, health policy

## Abstract

**Introduction:**

Person-centred care has become internationally recognised as a critical attribute of high-quality healthcare. However, the concept has been criticised for being poorly theorised and operationalised. Serious illness is especially aligned with the need for person-centredness, usually necessitating involvement of significant others, management of clinical uncertainty, high-quality communication and joint decision-making to deliver care concordant with patient preferences. This review aimed to identify and appraise the empirical evidence underpinning conceptualisations of ‘person-centredness’ for serious illness.

**Methods:**

Search strategy conducted in May 2020. Databases: CINAHL, Embase, PubMed, Ovid Global Health, MEDLINE and PsycINFO. Free text search terms related to (1) person-centredness, (2) serious illness and (3) concept/practice. Tabulation, textual description and narrative synthesis were performed, and quality appraisal conducted using QualSyst tools. Santana *et al*’s person-centred care model (2018) was used to structure analysis.

**Results:**

PRISMA (Preferred Reporting Items for Systematic Reviews and Meta-Analyses) flow data: n=12,446 studies screened by title/abstract, n=144 full articles assessed for eligibility, n=18 studies retained. All studies (n=18) are from high-income countries, and are largely of high quality (median score 0.82). The findings suggest that person-centred care encompasses the patient and family being respected, given complete information, involved in decision-making and supported in their physical, psychological, social and existential needs. The studies highlight the importance of involving and supporting family/friends, promoting continuation of normality and self-identity, and structuring service organisation to enable care continuity.

**Conclusion:**

Person-centred healthcare must value the social network of patients, promote quality of life and reform structurally to improve patients’ experience interacting with the healthcare system. Staff must be supported to flexibly adapt skills, communication, routines or environments for individual patients. There remains a need for primary data investigating the meaning and practice of PCC in a greater diversity of diagnostic groups and settings, and a need to ground potential components of PCC within broader universal values and ethical theory.

**Key questions**

**What is already known?**

- Person-centred care has become internationally recognised as a dimension of high-quality healthcare, promoted as a core competency of health workers, a key component of primary care and essential to achieving the Universal Health Coverage goals.
- Ongoing conceptual debates are attempting to determine what constitutes ‘person-centredness’ and how this concept can be understood and implemented in a variety of settings.
- Serious illness is especially aligned with the need for PCC; the complex clinical scenarios surrounding serious illness usually necessitate the involvement of significant others and depend on high-quality communication and joint decision-making to deliver care concordant with patient preferences, with recognition and management of clinical uncertainty.

**Key questions**

**What are the new findings?**

**What do the new findings imply?**

- Person-centred healthcare must value the social network of each patient, promote quality of life and personal goals not only health status improvement, and implement structural reforms to improve patients’ experience of interacting with the healthcare system.
- Health systems must be structured to enable sufficient availability and accessibility of health workers, and support staff to be able and willing to flexibly adapt skills, communication, routines or environments for individual patients.
- There is a need for primary data investigating the meaning and practice of PCC in a greater diversity of diagnostic groups and settings, particularly non-Western, low- and middle-income settings.
- There is a need to consider the theoretical underpinnings of PCC and to ground potential components within broader universal values and ethical theory.

##  Introduction

 Person-centred care has become internationally recognised as a dimension of high-quality healthcare.^1^ The Institute of Medicine describes quality care as that which is: “safe, effective, patient-centred, efficient, timely and equitable”.^2^ WHO policy on people‐centred healthcare highlights person‐centredness as a core competency of health workers, a key component of primary care, and essential to achieving the Universal Health Coverage goals.^3^,^6^

A variety of terms have been used to denote person-centred approaches. ‘*Patient*-centredness’ was first to gain prominence and aimed to challenge the reductionism of the biomedical model and stress the importance of psychosocial factors.^2 3^ Many moved towards use of the term ‘*person*-centredness’, suggesting this better articulates the holism of the ‘whole person’ and a broader conception of well-being.^7 8^ In recent years, the term ‘*people*-centredness’ has also gained prominence, emphasising a focus on “the whole person in their specific familial and community contexts”.^9^ Person-centred, patient-centred and people-centred care (PCC) all embody an approach that consciously adopts the perspectives of individuals, families and communities, respects and responds to their needs, values and preferences and sees them as participants in their own healthcare rather than just beneficiaries.^2 10^

Conceptual clarity is critical to the design, delivery and replication of successful innovations in care.^11^ Despite the global prominence of PCC as a goal of health systems, the approach suffers from a lack of clarity. Ongoing conceptual debates are attempting to determine what constitutes ‘person-centredness’ and how this concept can be understood and applied in a variety of contexts.^712^,^14^ While numerous conceptualisations of PCC are presented in existing literature,^815^,^21^ most do not appear to offer empirical origins or practical guidance on the implementation of PCC. The WHO *Global strategy on people-centred and integrated health services* recognises that there is not a single model of PCC to be proposed, but rather that it should be context-specific and that each country should generate its own evidence to enable appropriate, acceptable, feasible practice of PCC.^10^ It is currently unclear what evidence is available to model contextually-appropriate and culturally-appropriate PCC.

The need for a person-centred approach is particularly important in the context of serious illness. The complex clinical scenarios surrounding serious illness usually necessitate the involvement of significant others, high-quality communication and joint decision-making to deliver care concordant with patient preferences, with recognition and management of clinical uncertainty.^22^,^24^ As populations age, as infectious disease is better managed, and multimorbidity becomes more prevalent, serious health-related suffering associated with conditions such as cancer, chronic lung disease and dementia is rising fastest in low- and middle-income countries (LMICs).^25^ Serious illness is also a context in which delivering PCC can be more complex and may require more dimensions to a greater degree than for non-serious illness. Focussing specifically on serious illness is therefore a means of ‘stress testing’ generalist PCC theory and ensuring it captures ‘what matters’ in all diagnostic cases. A better understanding of PCC in the context of serious illness would have health-system-wide relevance for other less complex clinical scenarios.

This systematic review aims to aggregate and appraise the empirical evidence underpinning the concept and practice of PCC in the context of serious illness. Specifically, the objectives of the review are to answer the following questions:

1. What is the primary data underpinning conceptualisations and practice-based frameworks of ‘person-centredness’ in the context of serious illness?
2. What is the quality of this data?
3. What are the key constructs of PCC according to this data?

## Methods

This systematic review follows the Preferred Reporting Items for Systematic Reviews and Meta-Analyses (PRISMA) recommendations.^26^ The review protocol was registered prospectively with PROSPERO: https://www.crd.york.ac.uk/prospero/display_record.php?RecordID=139259 (registration number 139259).

### Definition of terms

To structure this review, literature was considered in line with two frequently cited definitions of PCC, one policy-led (using the term ‘*people*-centredness’) and one patient-led (using the term ‘*patient*-centredness’):

1. “An approach to care that consciously adopts the perspectives of individuals, families and communities and sees them as participants as well as beneficiaries of trusted health systems that respond to their needs and preferences in humane and holistic ways.” (WHO, 2015)^10^
2. “Care that is focussed and organised around people, rather than disease. Within this approach disease prevention and management are important but not enough to address the needs of person, family and community.” (International Alliance of Patients Organisations, 2007)^27^

These definitions informed the broad review search strategy.

Numerous terms exist relating to person-centred care, including patient-centred, people-centred, patient-directed and so forth. We acknowledge that these various terms have differences in their origins and connotations.^28^ However, as they overlap significantly and are often used interchangeably we chose to include all terms in the search strategy and analysis. When referring to this approach we chose to use the term ‘person-centred’. In agreement with Ekman *et al*^8^ and The Health Foundation,^29^ we take that view that the word ‘person’ avoids reducing the individual to a mere recipient of services and better highlights the whole human being with reason, preferences, needs and a social and cultural background.

The review focuses on serious illnesses in line with the following definition: “Serious illness carries a high risk of mortality, negatively impacts quality of life and daily function, and/or is burdensome in symptoms, treatments or caregiver stress. This includes conditions not advanced or high dependency/low function that carry a degree of clinical uncertainty” (Kelley *et al*, 2016).^30^

According to Kelley *et al*’s broadest definition of serious illness, serious medical conditions include: cancer (metastatic or hematological), renal failure, dementia, advanced liver disease or cirrhosis, diabetes with severe complications, amyotrophic lateral sclerosis, acquired immune deficiency syndrome, hip fracture, chronic obstructive pulmonary disease or interstitial lung disease if using home oxygen or hospitalised, and congestive heart failure if hospitalised for the condition.^30^

### Search strategy

The full search strategy is reported in online supplemental appendix A. The following databases were searched on 18 May 2020 with no date restrictions: Cumulative Index to Nursing and Allied Health Literature (CINAHL), Embase, MEDLINE, Ovid Global Health, PsycINFO and PubMed. Forward and backward reference chaining of included articles was performed.

We included free text search terms (title, abstract and keyword search) related to (1) person-centred care/patient-centred care, (2) serious illness and (3) concept or practice (the meaning of PCC or way in which PCC is enacted). Search terms were adapted to each database subject headings and ‘exploded’ terms. The specific serious conditions included were those listed by Kelley *et al*^30^ within their broad, operationalised definition of serious illness. Please see online supplemental appendix A for full list of search terms and example search strategy.

### Data collection and extraction

All potential references identified were exported to EndNote reference manager and deduplicated. The primary reviewer (AG) assessed the titles and abstracts against the inclusion and exclusion criteria (detailed in online supplemental appendix A). The full texts of remaining references were then similarly screened. Any reference for which inclusion was unclear was agreed through discussion with the secondary reviewer (KN) or adjudicated by a third reviewer (RH) if consensus was not reached. The following variables were extracted from retained studies into a common table: authors, year of publication, country, setting, aim and objectives, study design and methods, sample and main findings.

### Quality assessment

We applied Kmet *et al*’s Standard Quality Criteria^31^ to the primary data. The checklists (quantitative data n=14-items, qualitative data 10-items) score each criterion ‘yes’=2, ‘partial’=1 and ‘no’=0. Items deemed not applicable are excluded from the summary score, which ranges from 1 (highest) to 0 (lowest). Online supplemental appendix A further details the method to calculate scores. We did not exclude studies based on quality score. The primary reviewer (AG) assessed the quality of each study. The secondary reviewer (KN) also assessed the quality of n=5 of the studies and met with the primary reviewer thereafter to compare assessments, resolve any discrepancies and enable reflections to be applied to all other studies’ quality assessments.

For quantitative studies, Kmet *et al* propose a cut-off score of 0.75 as the threshold for including a paper in a review.^31^ As our goal was to assess data quality rather than exclude data failing to meet a quality threshold, we used Lee *et al*’s^32^ definitions for Kmet *et al*’s quality scores; strong (summary score of >0.80), good (summary score of 0.71 to 0.79), adequate (summary score of 0.50 to 0.70) and limited (summary score of <0.50). For qualitative studies, Kmet *et al* use a threshold of 0.55 for inclusion of a study into their systematic review,^31^ therefore we defined qualitative papers with scores of ≥0.55 as ‘adequate quality’ and ≤0.54 as ‘low quality’.

### Data analysis

Retained studies were analysed using narrative synthesis in line with Guidance on the Conduct of Narrative Synthesis in Systematic reviews.^33^ The preliminary synthesis was performed by tabulation, grouping and clustering.

To synthesise the extracted data the authors adopted a PCC model developed by Santana and colleagues^34^ (hereafter referred to as Santana model). The Santana model was selected to structure the analysis of retained studies as it provides comprehensive, practical guidance for implementation of PCC, explicitly linking this guidance to the Donabedian model for assessing healthcare quality.^35^ Santana *et al*’s model was generated through a narrative review and synthesis of evidence, recommendations and best practice from implementation case studies, as well as existing frameworks. However, besides the consultation of a patient representative, there is limited voice of patients and families informing the model. The model’s authors suggest validation of the framework with additional diverse patient perspectives and to identify any necessary revisions or additions.^34^

The components of the Santana model were used to construct an a priori coding frame for deductive analysis of the study findings retained in this systematic review (see online supplemental figure 1 for a priori coding frame). Findings that did not fit into the a priori frame were inductively coded into new codes. The primary reviewer (AG) coded the data using NVivo V.12 software, coding data that did not fit into the a priori frame into additional ‘Other’ nodes. The primary reviewer reviewed the contents of these ‘Other’ nodes throughout the analysis, generating new inductive codes where new themes appeared and revising or adding to these as more data was coded. New inductive codes were reviewed by the second and third reviewers (KN and RH), and discussed until consensus on new code meanings and labels was reached.

### Patient and public involvement

Patient and public involvement was not conducted as part of this review.

## Results

**Figure 1:**
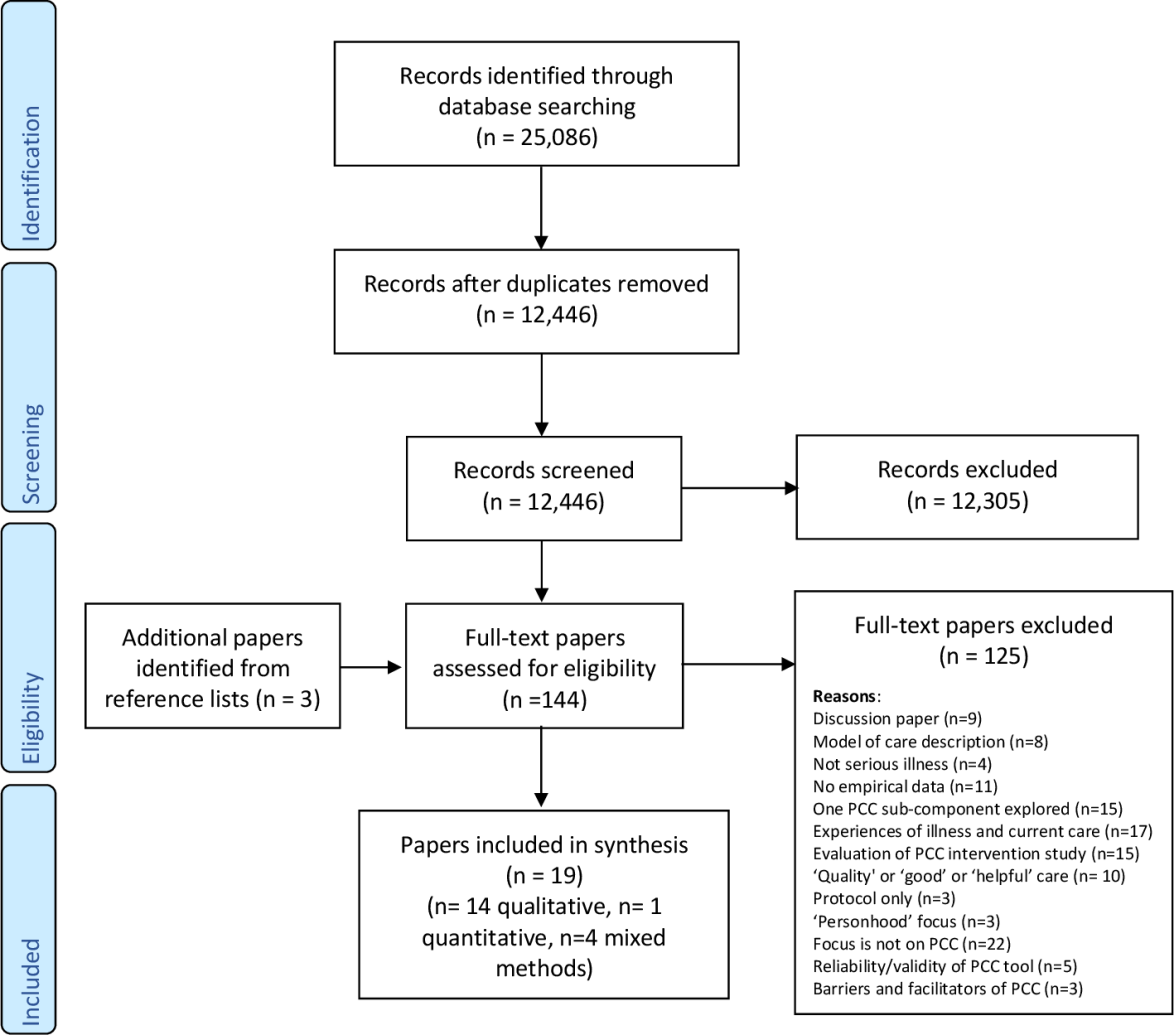
PRISMA (Preferred Reporting Items for Systematic Reviews and Meta-Analyses) 2009 flow diagram.

The search summary flowchart following PRISMA guidelines is presented in figure 1. The search yielded 12,446 references following deduplication, and 18 studies/n=19 papers^36^,^54^ were retained and synthesised in this review. The characteristics of included studies are summarised in box 1. Further detailed characteristics of each included study are presented in online supplemental table 1, with Kmet *et al*’s^31^ data quality score.

Box 1 **Characteristics of included studies**

**Countries and settings**

All retained studies (n=18/18) reported data from high-income, Western countries.

- The Netherlands (n=5/18)^36^,^40^
- Canada (n=3/18)^41^,^43^
- Australia (n=3/18)^44 45 49^
- USA (n=2/18)^46 50^
- UK (n=1/18)^47^
- Ireland (n=1/18)^53^
- Norway (n=1/18)^48^
- Sweden (n=1/18)^54^
- Germany (n=1/18 study reported in n=2/18 papers)^51 52^

Healthcare settings

- Hospital wards (n=5/18)^37 38 41 47 48^
- Residential aged care facilities (n=3/18)^44 45 54^
- Outpatient clinics (n=2/18)^36 50^
- Nursing homes (n=1/18)^53^
- Cancer centre (n=1/18)^42^
- Academic cancer institution (n=1/18)^43^
- Unknown/combination (n=5/18 studies reported in n=6/19 papers)^39 40 46 49 51 52^

**Diagnostic groups and healthcare professionals**

- Cancer (n=10/18 studies reported in n=11/17 papers)^3638 39 41^,^43 48^
- Dementia (n=4/18)^44 45 53 54^
- End-stage renal disease (n=1/18)^37^
- Palliative or end-of-life care (n=2/18)^40 46^
- Mixed diagnostic groups experiencing acute care (n=1/18)^47^

**Participant groups included**

- Healthcare professionals (n=14/18 studies reported in n=15/18 papers)^3739^,^47 49 51^
- Patients (n=10/18)^36^,^3942 44 48^
- Caregivers (n=3/18) studies included^42 44 49^
- Volunteers working in palliative care (n=1/18)^40^

**Study designs**

Qualitative designs (n=13/18):

- Semi-structured interviews (n=11/18 studies reported in n=12/19 papers)^3638 43^,^49 51^
- Focus groups (n=2/18)^43 47 50^
- Case studies (interview and observation) (n=1/18)^41^
- Mixed qualitative methods (posters and interviews, n=1/18)^42^ interviews and focus groups, (n=1/18)^49^

Quantitative design (n=1/18):

- Survey (n=1/18)^54^

Mixed-methodology designs (n=4/18):

- Q methodology (n=2/18)^37 40^
- Questionnaire (n=1/18)^38^
- Delphi method (n=1/18)^39^

**Term used to refer to the PCC approach**

- Patient-centred care (n=8/18)^36^,^4048^
- Person-centred care (n=7/18)^42 44 45 47 49 53 54^
- Patient-centred and family-centred care (n=1/18)^43^
- Client-centred care (n=1/18)^46^
- Individualised integrative care (n=1/18 reported in n=2/18 papers)^51 52^
- Interprofessional patient-centred care (n=1/18)^41^

**Kmet Data Quality Scores**

Range=0.35 to 0.95 (possible range: 0 to 1)

Median=0.82

Qualitative studies and qualitative component of mixed-methods studies (n=17/18):

- n=17 scored ≥0.55 (adequate quality)
- n=1 scored ≤0.54 (low quality).

Quantitative studies and quantitative component of mixed-methods studies (n=5/18):

- n=4 scored >0.80 (strong)
- n=1 scored 0.71–0.79 (good)

**Summary of aims and research questions of studies retained in this review**

- n=8/18 studies included an objective to investigate what is understood by the term PCC or what PCC should consist of in practice.^3740 42 44^,^48^
- n=3/18 studies focused on patients’ experiences and expectations of care in relation to predetermined ideas of PCC components.^36 49 50^
- n=2/18 studies aimed to develop PCC indicators.^38 39^
- n=2/18 studies (reported in n=3/17 papers) aimed to investigate how teams that identify as providing PCC practice their care.^41 51 52^
- n=2/18 studies aimed to investigate clinicians’ knowledge and attitudes towards PCC.^43 53^
- n=1/18 study aimed to identify the organisational, environmental, resident and staff variables associated with aged care units with higher perceived levels of PCC.^54^

### Synthesis of included studies’ findings

#### Patient-family-provider relationship

Overall, the findings suggest that PCC encompasses empowerment of both the patient and their family by being respected,^40 41 48 50 53^ listened to,^36 37 47 48^ understood,^47^ given honest, complete and comprehendible information^3637 39^,^41 43 48 49^ and by being engaged in treatment decisions and all decisions that affect their daily life and care.^36 37 42 46 48 50 51^ This requires collaborative, trusting relationships to be developed between patients, families and clinicians,^4047 49^,^51^ which rely on clinicians’ communication skills,^39 43 49^ attitude^41^ and demonstrable compassion,^42^ for example, by comforting nervous patients.^36^ The studies highlighted specific patient information needs, for example, using diagrams or drawings to aid comprehension, using accessible language, providing information about the possible course of the disease and information about the treatment option of ‘no active therapy’.^38 49^ It was also raised that patients should be given the necessary information, education and support to enable self-management.^39^

A further dominant theme was the importance of involving and supporting the patient’s family, friends or significant others,^36 44 46 47 49^ although some patients may deem this a lesser priority.^37 40^

In addition to physical symptom control, the studies suggest patients must also be supported in their psychological, social and spiritual needs,^39 40 45 49 51 52^ with great attention to all needs and aspects of care that are important to the person.^36 42 46 47^ Sufficient time^51 52^ and availability of staff^41^ was identified as crucial to address these needs.^54^ This also requires flexibility and willingness to adapt skills, routines or environments for individual patients.^44 46^

Several studies’ findings placed weight on promoting autonomy, continuation of self and normality and enabling patients to participate in life.^44 45 52 53^ This was particularly highlighted in studies focussed on dementia patients and nursing homes,^44 53^ where a dementia-friendly physical environment was also deemed important.^54^

#### Organisational level requirements

On an organisational level, PCC was reported to demand a shared philosophy of care,^54^ satisfactory leadership, support from colleagues and continuing education and mentorship of staff.^54^ PCC was seen as requiring interdisciplinary collaboration,^51 54^ and consistency and regularity in collaboration of all members of a care team.^41^ Furthermore, all staff (not only front-line) were deemed responsible for providing person-centred care.^42^ Included studies highlighted the importance of the coordination and continuity of patient care^44 49^ and of streamlining care delivery,^43^ for example, by having nursing staff provide additional teaching following the physician visit,^43^ or appointing each patient a care coordinator.^37 39 49^ Studies also indicated the importance of enhancing accessibility of healthcare services and considering logistical barriers, such as lack of transport or financial resources.^49^

#### Complementary findings across participant groups, across countries and across PCC terms

There were no clear discrepancies between the findings of studies incorporating patient participants, caregiver participants or healthcare professional participants. The heterogeneity of studies did not permit analysis to determine difference between countries or regions. However, the study conducted with indigenous Australian populations reported study-specific findings such as the high financial burden of accessing care and the importance of feeling ‘culturally safe’ within the healthcare system.^49^ There was also no evidence of consistent differences between findings from studies using different terms within the PCC consortium, that is, patient-centred care, patient-centred and family-centred care, client-centred care and so on. Based on the WHO definition of ‘people-centredness’, we hypothesised that this term has conceptual differences to person-centredness and patient-centredness and wished to investigate what these may be. However, as none of the retained empirical studies used this term we did not have the opportunity to investigate this.

### Domains of Santana model supported by included studies’ data

The data from included studies largely supported the Santana model components (online supplemental table 2), providing more detail about the specific meanings of subdomains, and suggesting relationships between concepts. This is particularly the case for many of the model’s Process dimensions which saw numerous corresponding data codes, for example, *Being responsive to preferences, needs and values; Sensitivity to emotional or psychosocial needs; Sharing information; Shared decision-making*.

*Understanding patient within his or her unique psychosocial or cultural context* is an example of a Santana model domain that is better specified through the included studies’ findings. Findings related to this domain suggest that clinicians should show interest in the person as a whole and gain an understanding of their psychological and emotional health, spiritual and existential issues, living conditions, financial situation, social support system, culture, personal identity and daily routines and activities. This knowledge should then be translated into tailored care, perhaps providing emotional support from nurses, referring to appropriate specialists, considering patient convenience and resource availability when ordering investigations and initiating conversations and activities that may be meaningful to a particular patient.^3638 39 42^,^47 49 51 52^

### Domains of Santana model left unpopulated by included studies’ data

Table 1 presents domains of the Santana model for which no corresponding study data was found. Predominantly, the Structure components of the Santana model were unpopulated by findings from the 16 studies. This includes domains such as “S3. Co-designing the development and implementation of health promotion and prevention programs”, and “Spiritual and religious spaces”. “P2b. Providing resources” was the only Process domain to be left unpopulated by the data. Outcome dimensions “O2b Patient-Reported Experiences (PREMs)” and “O2c. Patient-Reported Adverse Outcomes (PRAOs)” were left with no corresponding findings from included studies.

**Table 1:** Santana model domains with no assigned codes from included studies:

Structure	“S1a. Core values and Philosophy of the organisation” subdomains: “Vision and mission” “Patient and healthcare provider rights” “S1b. Establishing operational definition of PCC” subdomains: “Consistent operational definitions” “Common language around PCC” “S2. Co-designing the development and implementation of educational programs” subdomains: “Standardised PCC training in all healthcare professional programs” “Professional education and accrediting bodies” “S3. Co-designing the development and implementation of health promotion and prevention programs” and all sub-domains “S4a. Ensure resources for staff to practice PCC” and subdomain: “Provide adequate incentives in payment programs; celebrate small wins and victories” “S5. Providing a supportive and accommodating PCC environment” subdomains: “Collaborate with and empower patients and staff in designing healthcare facilities” “Facility that prioritise the safety and security of its patients and staff” “Spiritual and religious spaces” “Patient-directed visiting hours” “S6. Developing and integrating structures to support health information technology” and all subdomains “S7. Creating structures to measure and monitor PCC” and subdomain: “Co-design and develop framework for measurement, monitoring and evaluation”
Process	“P2b. Providing resources”
Outcome	“O2b Patient-Reported Experiences (PREMs)” and subdomain: “Recommendation or rating of hospital, healthcare provider” “O2c. Patient-Reported Adverse Outcomes (PRAOs)” and subdomains: “New or worsening symptoms” “Unanticipated visits to healthcare facilities”

### Model adaptation: evidence additional to Santana model domains

Additional units of meaning arose from the included studies that are currently lacking in the Santana model: *Family and friend involvement and support, Promoting continuation of normality and self-identity and Structuring service organisation to enable continuity of care and patient navigation*. Table 2 presents these inductively-identified additional themes with examples of corresponding codes from supporting studies. Table 3 presents an adapted version of the Santana framework incorporating these additional themes.

**Table 2:** Inductively-identified themes additional to Santana model with corresponding codes (see online supplemental table 2 for full table of studies’ findings deductively mapped onto Santana model and inductively mapped onto additional themes)

Inductively-identified themes additional to Santana model	Number of supporting studies	Examples of corresponding codes from supporting studies
Family and friend involvement and support Involving family/friends in information-sharing and decision-making Respecting the opinions and worries of friends/family Providing family/friends with opportunities to ask questions Addressing the needs of family/friends	11 36,4042 44 46 47 49 51	*“Involving the family is a massive part of person-centred care, as their family know everything about them; they just know them inside out…” (Registered nurse 4*). (Ross et al, 2015, p1228; Quality score 0.8)^47^ *Welcoming family was described as developing and maintaining trust in that the staff would actively communicate changes and signiﬁcant events to the family; so that they did not have to constantly seek out information by making phone calls or asking the staff when they visited. Welcoming family was also described by staff as creating opportunities for beneﬁcial teamwork, so that the family’s unique knowledge about the person with dementia could be incorporated into care plans*.^44^ (Edvardsson et al, 2010, p2614; Quality score 0.8)^44^ *Respondents in both views state that it is important to attend to the preferences of patients first, and to those of the family thereafter…‘Of course there are some meetings involving the whole family, but ultimately, it is the patient who decides and not the family’ (respondent 13*).^40^ (Galekop et al, 2019, p4; Quality score 0.95 (qualitative), 0.85 (quantitative))^40^ *The involvement and respect to the opinions and worries of friends and family was considered very important for the majority of patients*.^36^ (Bisschop et al, 2017, p2250; Quality score 0.85)^36^ *(Suggested patient-centred cancer care indicators): Family and friends had opportunities to ask the specialists questions; Family and friends had opportunities to ask the nurses questions*.^38^ (Ouwens et al, 2010, p126; Quality score 0.65 (qualitative), 0.78 (quantitative))^38^ *C2. The care provider should gather information on the psychosocial and emotional health status of family and friends of the patient and adequately refer to specialists, depending on the diagnosed problems*. (One of final set of approved quality indicators),^39^ (Uphoff et al, 2011, p35; Quality score 0.8 (qualitative), 0.88 (quantitative))^39^ *It was noted that carers undergo sustained periods of dealing with multiple stressors, combined with a lack of attention to their needs and their welfare and little-to-no follow-up, and that this contributed to a sense of being disregarded once the person being cared for had passed away or had reached a less acute stage. “…the carer is the one that carries the load. You know they’re the ones that are looking after the sick person as well as trying to manage family.” (103 Both*).^49^ (Green et al, 2018, p8; Quality score 0.85)^49^
Promoting continuation of normality and self-identity Support for participating in regular personal life activities Providing meaningful activities for inpatients	8 3738 44,46 49 51 52	*Themes and goals centred on understanding “disease as a path/journey”; to “live as a human being” by participating in life despite the disease; to regain activity, control and hope*.^51^ (Kienle et al, 2016, p483, Quality score 0.9)^51^ *The overarching themes of doctors were to help patients live with the disease and ﬁnd their own way through it; to encourage them to participate in life and regain autonomy and a sense of control and self-efficacy.*^52^ (Kienle et al, 2018, p128; Quality score 0.9)^52^ *For instance, some patients were not afraid of death but rather of losing certain sensibilities or fine cognitive functions: “For instance a patient with advanced oesophagus carcinoma wished to write several publications; he had a spiritual orientation and rejected chemotherapy because he feared cognitive impairments and emotional constraints; with regular intralesional mistletoe extract injections the oesophageal stenosis reopened, the patient could eat and kept well for a substantial time with a good quality of life and pursued his writing and publishing activities.”(Gastroenterologist),*^51^ (Kienle et al, 2016, p482, Quality score 0.9)^51^ *Individually targeted activities were described not only as providing a meaningful content to the day, but also as a means in reafﬁrming the residents as individual persons who were able to do the things they enjoyed. Family and staff further described that such activities preferably were adapted to the individual person’s ability so that their self-esteem could be boosted by the successful completion of activities, rather than feeling defeated and demoralised by being expected to undertake something that was beyond their capability*.^44^ (Edvardsson et al, 2010, p2615; Quality score 0.8)^44^ *Two of the participants suggested that in their client-centeredness they ‘manipulated’the situation to enable choices, but ones that further occupationally engaged their clients. One participant expressed that the development of the emotional climate or helping people at the end of life engage in full expression of themselves was client-centred and family-centred*.^46^ Pizzi, 2015, p446; Quality score 0.65)^46^
Structuring service organisation to enable continuity of care and patient navigation Simplification of care pathways to ease patient navigation Appointment system structured to allow patients to see same professionals over time Structures enabling flexibility in service delivery and care practice. Establishing cooperation pathways across specialisms and institutions	10 36,3841 43 44 46 47 49 51	*Using multidisciplinary clinics to decrease wait times and patient anxiety between specialist referrals; Having nursing staff provide additional teaching following the physician visit*. (Physician identified patient- and family-centred strategy pertaining to streamlining care delivery.)^43^ (Nguyen et al, 2017 (online supplemental table 2); Quality score 0.65)^43^ *The complex organisation of services could also affect the experience of care. Another family member summarised this: “The system(the oncology clinic) is so complicated that it’s like swimming in molasses. (Family member 3*)^41^ (Bilodeau et al, 2015, p109; Quality score 0.7)^41^ *The most prominent negative experiences noted were due to seeing different doctors at subsequent appointments: “We had an appointment with our doctor, but then we received a message that a new doctor was scheduled to help us that day. We really did not like that, especially because he had to tell us new test results and the prognosis. There was no explanation; they only told us our regular doctor was absent.” (Respondent 12*)^36^ Bisschop et al, 2017, p2250; Quality score 0.85)^36^ *Mostly in data from staff and family, it emanated that to be person-centred, aged care facilities need to have ﬂflexible routines adapted to the person with dementia’s needs rather than the needs of staff, especially in relation to stafﬁng, care tasks and activities.*^44^ (Edvardsson et al, 2010, p2616; Quality score 0.8)^44^ *Some doctors worked in a cancer centre…the others cooperated with oncologists, surgeons, radiotherapists, and other relevant specialists, often referring patients to each other. This cooperation was usually described as positive: “These centres know me all for long… they know that I know exactly what they do… it functions well” (Paediatrician*).^51^ (Kienle et al, 2016, p488, Quality score 0.9)^51^

**Table 3:** Adapted Santana framework incorporating additional themes from the empirical evidence (presented in bold text)

Structure	Process	Outcome
S1. Creating a PCC culture subdomain S1a. Core values and philosophy of the organisation S1b. Establishing operational definition of PCC S2. Co- designing the development and implementation of educational programs Standardised PCC training in all healthcare professional programs **Training in holistic perception of human organism** **Training for non-clinical staff in providing compassionate and co-ordinated PCC** S3. Co- designing the development and implementation of health promotion and prevention programs S3a. Collaboration and empowerment of patients, communities and organisations in design of programs S4. Supporting a workforce committed to PCC S4a. Ensure resources for staff to practice PCC ** S4b. Ensure strong team leadership around PCC** S5. Providing a supportive and accommodating PCC environment S5a. Designing healthcare facilities and services promoting PCC S5b. Integrating organisation-wide services promoting PCC S6. Developing and integrating structures to support health information technology Common e-health platform for health information exchange across providers and patients S7. Creating structures to measure and monitor PCC performance Co-design and develop framework for measurement, monitoring and evaluation **S.8 Structuring service organisation to enable continuity of care and patient navigation** ** S8a. Simplification of care pathways to ease patient navigation** ** S8b. Appointment system structured to allow patients to see same professionals over time** ** S8c. Structures enabling flexibility in service delivery and care practice**. ** S8d. Establishing cooperation pathways across specialisms and institutions**	P1. Cultivating communication P1a. Listening to patients P1b. Sharing information P1c. Discussing care plans with patients P2. Respectful and compassionate care P2a. Being responsive to preferences, needs and values P2b. Providing supportive care ** P2c. Promoting continuation of normality and self-identity** **Support for participating in regular personal life activities** **Providing meaningful activities for inpatients** P3. Engaging patients in managing their care Co-designing care plans with patients P4. Integration of care Communication and information sharing for coordination and continuity of care across the continuum of care Between healthcare providers Referrals to specialist Discharge communication Providing access to information and resources Cooperation across specialisms and institutions **P5. Family and friends’ involvement and support** ** P5a. Involving family/friends in information-sharing and decision-making** ** P5b. Addressing the needs of family/friends**	O1. Access to care O1a. Timely access to care Components O1b. Care availability O1c. Financial burden O2. Patient-Reported Outcomes (PROs) O2a. Patient-Reported Outcomes Measures (PROMs) O2b. Patient-Reported Experiences (PREMs) O2c.Patient-Reported Adverse Outcomes (PRAOs)

Specifically, *Family and friend involvement and support* was described as: inviting the patient to bring someone to appointments,^39^ establishing conversation with family/friends;^42^ involving family/friends in information-sharing and decisions regarding the patient’s care;^37^ providing family/friends with opportunities to ask specialists and nurses questions;^38^ respecting the opinions and worries of friends/family;^36^ acknowledging family/friends in their role as carer for the patient;^37 44^ and involving family/friends at all stages including long-term care, treatment and follow-up.^38^ Being involved was deemed to avoid feelings of anxiety among family^44 49^ and aid the patient emotionally, practically and in understanding and reflecting on information provided by clinicians.^49 51^ This domain of PCC also requires healthcare professionals to pay attention to the needs of family/friends of the patient,^37 46 49^ including providing accommodations in or near the hospital during treatment if possible,^37 49^ and gathering information on the emotional health of family/friends and referring to specialists as appropriate.^39^ It is worth noting that some patients and professionals may place this need as a low priority compared with other PCC domains.^37 40^

*Promoting continuation of normality and self-identity* was discussed as requiring encouragement and enablement of persons with serious illness to participate in life despite the disease, and to regain a sense of control and self-efficacy.^51 52^ This requires the clinician to consider a patient’s life goals and self-identity when discussing care and treatment options.^51^ For long-term inpatients, particularly those with dementia, arranging and enabling meaningful activities was also viewed as a critical part of PCC. Creating individually targeted activities were described not only as providing a meaningful content to the day, but also as a means in reafﬁrming the residents as individual persons who were able to do the things they enjoyed.^44^

*Structuring service organisation to enable continuity of care and patient navigation* encapsulates a collection of studies’ findings highlighting the importance of streamlining and easing patient navigation, ensuring continuity of care and simplifying the process of multi-specialist care. Suggestions for enabling this included appointing each patient a care coordinator or liaison officer,^37 41 49^ ensuring patients see the same professionals over time^36 41 44^ using multidisciplinary clinics to decrease wait times and patient anxiety between specialist referrals,^43^ and arranging for nursing staff to provide additional information or education following a physician visit.^43^

## Discussion

This review has revealed that a number of different constructs underpin the meaning and practice of PCC in the research evidence. These include patient and family empowerment and autonomy through respectful communication, appropriate information sharing and shared decision-making, addressing psychological, social, spiritual and cultural needs and enhancing coordination and continuity of care. The findings of this review indicate that person-centred healthcare must value the social network of each patient, and should promote quality of life and personal goals, not only health status improvement. This implies that person-centred health systems should be structured with flexible health workforce capacity and support staff to adapt skills, communication, routines or environments for individual patients and their families.

The studies’ findings largely validate the domains of the Santana framework of PCC, supporting their importance and providing more detail about specific meanings and subcomponents. The empirical findings of included studies also highlight new PCC themes additional to the Santana model. In focussing on serious illness, this review provides insights into the meaning of PCC that other, less severe conditions may not draw attention to.

The additional theme from included studies’ findings*: Family and friend involvement and support*, is in line with several other prominent conceptualisations of PCC.^2 16 55^ It particularly aligns with conceptualisations that focus on ‘*people*-centred’ care, such as that by the WHO, bringing attention to the health of people within their full social circles and communities.^56 57^ The vast majority of everyday care is often undertaken by patient’s families and social networks. Enabling families and friends to be active participants in a patient’s healthcare should therefore rightly be a key goal of person-centred health systems reform.

Included studies also indicate PCC as enabling patients to continue to participate in daily life and meaningful activities, promoting continuation of self, personal identity and normality. This finding emphasises that patients’ highly value quality of life and continuation of their normal lives, not only health status improvement. This supports the idea that PCC involves striving to avoid damage to personal identities that the person values,^58^ and ties into findings from research with frail populations showing patients value care that supports ‘getting back to normal’ or ‘finding a new normal’.^59^ This finding also overlaps with a dimension of Mead and Bower’s patient-centredness framework: the ‘patient-as-person’, which places focus on the individual’s experience of illness and the impact of illness on the individual’s life or sense of self.^15^

The third additional theme*: Structuring service organisation to enable continuity of care and patient navigation,* places particular weight on the organisational and structural reforms that are needed to enable person-centred, care-continuity processes. It highlights that PCC requires not only aspects of the clinician–patient interaction to reform, but also the experience the patient has in interacting with the wider healthcare system. Continuity of care has been presented within other prominent conceptualisations of PCC^17 17 18 55 55^ however the specific structural features needed to enable this are rarely discussed. This review’s findings point towards some practical steps for achieving this, such as appointing each patient a care coordinator or arranging for nursing staff to provide additional teaching following a physician visit.

### Strengths and limitations

The literature search conducted was comprehensive, considered numerous synonyms for PCC and involved no country or year of publication restrictions. This review also benefitted from interdisciplinary, multinational co-authors, allowing a range of perspectives and cultural viewpoints to inform the analysis and discussion. However, the review does suffer some limitations. First, only peer-reviewed studies published in English were included. Second, the review research questions and search strategy relating to ‘practice’ may have contributed to the lack of supporting data for structure and outcome domains of the Santana model. Third, only publications that included the term ‘person-centred’ (or synonym) were included. Research has certainly been conducted in non-Western LMICs that could inform models of PCC, for example, studies investigating ‘good communication skills’ or ‘empathetic care’. However, searching terms related to, in addition to near synonyms of, PCC would have deemed this review unfeasible. Our aim was to understand PCC as it is currently described.

## Conclusions; implications for PCC research, policy and practice

This review indicates that there is a stark absence of theoretical models of PCC for serious illness that are grounded in empirical data. Future research should aim to generate theoretically-underpinned empirical frameworks for clinicians and policy makers on how to implement PCC through relevant, appropriate healthcare delivery.

It would also be insightful for future studies to further investigate the aforementioned PCC domains additional to the Satana model to validate whether these domains should constitute PCC components, and if so, what the specific, operationalisable actions within those components should be. One particular additional theme, *Involving and supporting the patient’s family and friends*, unsurprisingly surfaced most clearly in studies that included caregivers as participants (n=3). This highlights the importance of including this participant group in further empirical studies.

The included studies add depth and detail to existing Santana model domains, such as: *Understanding patient within his or her unique psychosocial or cultural context*. The findings related to this domain recognise that much of health is determined outside the clinic by social situations beyond the patient–clinician interaction, such as education, employment, income, housing, social support and gender.^60^ Acknowledging and addressing these social determinants of health are critical to delivering PCC. Healthcare professionals must be given the support, tools and structures to actively engage with these social determinants of a person’s health and illness. However, this finding also raises the wider question of where the responsibility of PCC lies and how much of this rests with the individual clinic and clinician. Certain socially determined aspects of patient health can be positively influenced by a healthcare professional, others cannot. Consideration is needed about how and when clinicians should go beyond the clinic, and how to involve any external actors in contributing towards better patient health outcomes.^61^ We must reflect on how a practice-based theory of PCC should sit within the broader socio-economic and cultural environment in which a health system operates.

Included studies also strongly support Santana model domains revolving around information sharing, shared decision-making and clinicians taking the time to properly understand each patient’s needs. This reaffirms the importance of in-depth holistic assessment of the patient and the need to empower patients and families through health literacy, equipping them with the knowledge to make informed decisions.^62^

Several Outcome and Structure components of the Santana model were left unsupported by findings from the studies. This is not to say that those subdomains are unimportant, but that evidence to support them is lacking, and that patients, caregivers and professionals are most immediately exposed to, and concerned with, discussing processes. Future primary research with healthcare managers or policy makers should specify important structural and outcome domains. However, we could also perhaps infer that patients and caregivers facing serious illness are as, or even more*,* concerned with the quality of *processes* than with the outcomes which are most often the focus of healthcare improvement efforts. This suggests we should value process improvements as we value outcome improvements and should value the processes of person-centred care in and of themselves rather than just as a means to a series of outcomes. This supports ethical arguments that we should recognise the intrinsic, not just instrumental, value of PCC, and should pursue it as a valued quality and ethical domain in its own right.^13 58^

The lack of study findings corresponding to some Structure components of the Santana model may also be a result of the lack of diversity in settings and diagnostic groups of included studies. The components left unpopulated by the studies’ findings appear to be those less relevant among the diagnostic groups and high-income settings of included studies. For example, *Facility that prioritises the safety and security of its patients and staff* is less likely to be voiced as a concern in high-income settings with lower rates of violent crime and civil unrest. *Health promotion* is an element of PCC that seems less poignant in cases of patients with end-of-life cancer and dementia; this topic may be of greater relevance in other serious conditions that are more responsive to lifestyle factors, such as chronic obstructive pulmonary disease. More empirical work is needed to confirm whether these components are of importance, what these components consist of and how they should be operationalised in day-to-day practice. This empirical investigation would be most insightful if conducted in a diverse range of contexts within which these components are likely to be more relevant.

PCC is an approach that evolved from high-income countries, and African theorists have questioned the relevance of Eurocentric conceptualisations and noted the absence of data to understand the meaning, feasibility and acceptability of PCC in non-Western LMICs.^63^ This is unsurprising given existing biasses in healthcare research towards high-income countries, and limited resources and platforms for LMICs to conduct and promote this research. In the context of fewer resources, PCC may also be mistakenly perceived as a ‘nice-to-have luxury’ rather than a ‘need-to-have necessity’ and may be challenging to promote in settings with a history of disease-specific, vertical programmes. However, the lack of diversity in study countries raises questions about how both Santana model domains and additional themes could be conceptualised and operationalised globally, in a diversity of settings. Successful enactment of person-centred care would require a multitude of contextual and cultural factors to be considered and accommodated. For example, as Markus and Kitayama^64^ discuss, the dominant construal of *self* differs between Western and other contexts. Western notions of the ‘self’ are that of an individual independent agent, while in most non-Western societies the ‘individual’ is more integrated with significant others. A patient with more interdependent views of self may be highly concerned with harmonising relationships and views. This has very real implications for the clinician–patient interaction and how to best practice involvement and support of a patient’s family and wider social network. Data from more individualistic cultures, such as that from the included Galekop *et al* study,^40^ may suggest that *‘*there are some meetings involving the whole family, but ultimately, it is the patient who decides and not the family*’*. In a more collectivist culture, however, great importance may be placed on collective decision-making and the impacts of illness on a person’s network,^65^ and thus, person-centred care would need to enable this. We must carefully consider the underlying values and determinants of culture in order to ensure cultural sensitivity in PCC theory.^58 66^ A global theory of PCC and resulting policy would need to accommodate different beliefs and worldviews and centre around a common set of human values.

## Supplementary material

10.1136/bmjgh-2020-003330 online supplemental appendix 1

10.1136/bmjgh-2020-003330 online supplemental table 1

10.1136/bmjgh-2020-003330 online supplemental table 2

## Data Availability

All data relevant to the study are included in the article or uploaded as supplementary information. This paper is a systematic review and does not report novel primary data.
