# Supplementary material for: The empirical evidence underpinning the concept and practice of person-centred care for serious illness: a systematic review
Source: BMJ Glob Health. 2020 Dec 10;5(12):e003330. doi: 10.1136/bmjgh-2020-003330 (PMC7733074; doi:10.1136/bmjgh-2020-003330)
Supplement: online supplemental appendix 1 [file bmjgh-2020-003330supp001.pdf]

Appendix A: Methods

Table 1 lists the free text search terms used for the literature search strategy.

Table 1. Search terms (title, abstract, and keyword search)

Concept 1		Concept 2		Concept 3
person-cent?red*; patient-cent?red*; people-cent?red*; patient-led; patient-directed; patient-focused; patient-oriented; client-cent?red; "values-based care" ; family-cent?red; relationship-cent?red; "individuali?ed care"	AND	"serious illness*"; "serious condition*"; "serious disease*"; "advanced illness*"; "advanced condition*"; "advanced disease*"; "incurable illness*"; "incurable condition*"; "incurable disease*"; palliative<br><br>cancer; "renal failure"; dementia; "chronic obstructive pulmonary disease"; "advanced liver disease"; "serious diabetic complications"; "amyotrophic lateral sclerosis"; "acquired immune deficiency syndrome"; "hip fracture"; "interstitial lung disease"; "congestive heart failure"; "chronic heart failure"	AND	concept*; practice*; framework; model; theory;<br><br>meaning; understand*; experience*; needs; views; perspective*; preference*; priorities

Table 2 details an example search strategy carried out on Embase database.

Table 2. Example search strategy

**OVID Search Strategy**<br>Database: Embase <1974 to 2019>
1.person-cent?red*.mp.<br>2. patient-cent?red*.mp.<br>3. people-cent?red*.mp.<br>4. patient-led.mp.<br>5. patient-directed.mp.<br>6. patient-focused.mp.<br>7. patient-oriented.mp.<br>8. client-cent?red.mp.<br>9. "values-based care".mp.<br>10. family-cent?red.mp.<br>11. exp family centered care/<br>12. relationship-cent?red.mp.

13. "individuali*ed care".mp.
14. 1 or 2 or 3 or 4 or 5 or 6 or 7 or 8 or 9 or 10 or 11 or 12 or 13
15. "serious illness*".mp.
16. "serious condition*".mp.
17. "serious disease*".mp.
18. "advanced illness*".mp.
19. "advanced condition*".mp.
20. "advanced disease*".mp.
21. "incurable illness*".mp.
22. "incurable condition*".mp.
23. "incurable disease*".mp.
24. palliative.mp.
25. exp palliative therapy/
26. exp palliative nursing/
27. cancer.mp.
28. "renal failure".mp.
29. exp kidney failure/
30. dementia.mp.
31. exp dementia/
32. "chronic obstructive pulmonary disease".mp.
33. exp chronic obstructive lung disease/
34. "advanced liver disease".mp.
35. "serious diabetic complications".mp.
36. "amyotrophic lateral sclerosis".mp.
37. exp amyotrophic lateral sclerosis/
38. "acquired immune deficiency syndrome".mp.
39. exp acquired immune deficiency syndrome/
40. "hip fracture".mp.
41. exp hip fracture/
42. "interstitial lung disease".mp.
43. exp interstitial lung disease/
44. "congestive heart failure".mp.
45. exp congestive heart failure/
46. "chronic heart failure".mp.
47. 15 or 16 or 17 or 18 or 19 or 20 or 21 or 22 or 23 or 24 or 25 or 26 or 27 or 28 or 29 or 30 or 31 or 32 or 33 or 34 or 35 or 36 or 37 or 38 or 39 or 40 or 41 or 42 or 43 or 44 or 45 or 46
48. 14 and 47
49. concept*.mp.
50. concept analysis/ or thinking/
51. practice*.mp.
52. evidence based practice/
53. practice guideline/
54. framework.mp.
55. conceptual framework/
56. model.mp.
57. theoretical model/
58. theory.mp.

59. theory/
60. meaning*.mp.
61. understand*.mp.
62. experience*.mp.
63. needs.mp.
64. views.mp.
65. perspective*.mp.
66. preference*.mp.
67. priorities.mp.
68. 49 or 50 or 51 or 52 or 53 or 54 or 55 or 56 or 57 or 58 or 59 or 60 or 61 or 62 or 63 or 64 or 65 or 66 or 67
69. 14 and 47 and 68
****

Table 3 presents details of the inclusion and exclusion criteria used to screen studies.

Table 3. Inclusion and exclusion criteria

	**Inclusion criteria**
a.	Papers were included if they present primary data of any study design reporting evidence on the meaning, preferences or practices of person-centred care from the perspective of adult patients with serious illness, their family members, caregivers or any individuals who work with patients with serious illness or are responsible for management or policy making for any healthcare settings that provide care to those with serious illness.
b.	Papers were included if 1) investigation of the meaning or practice of PCC is included in the aims or objectives of the study, 2) data concerning meaning or practice of PCC is presented in the results section of the paper, as we understand that this finding may result from a related aim.
c.	Qualitative, quantitative and mixed-method studies of any study design were considered: quantitative studies of all descriptive, correlational, quasi-experimental or experimental designs were included, and qualitative studies of phenomenological, ethnographic, grounded theory, historical, case study, or action research design will be included.
d.	Studies involving the following types of participants were included: adult patients with serious illness (18 years and older); their family members or friends; their caregivers; individuals who work with patients with serious illness (these include general practitioners, specialist doctors, hospital and community nurses, patient representatives, medical students, social workers, and all other clinical staff interacting with such patients); individuals responsible for management or policy making for any healthcare settings that provide care to those with serious illness. Patients with serious illness were restricted to those as defined by Kelley et al [1].
e.	Studies conducted anywhere in the world (low, middle and high-income countries) were included.
f.	Studies published in English were considered for inclusion in this review.
g.	Studies of any publication date were considered for inclusion; no date restriction was applied.

1] Kelley, A.S., et al., *Identifying Older Adults with Serious Illness: A Critical Step toward Improving the Value of Health Care.* Health Serv Res, 2017. **52**(1): p. 113-131.

Exclusion criteria
a. Published literature other than primary studies (i.e. review articles, books, policy or commentary papers).<br>b. Studies focusing on intervention outcomes or effectiveness.<br>c. Studies investigating whether patients have a preference for ‘person-centred care’ that do not provide any investigation of the meaning or practice of this concept.<br>d. Studies investigating barriers and facilitators for PCC, that do not include any investigation of the meaning, preferences or practices of person-centredness. This review is focused on understanding what PCC means, consists of, and looks like in practice rather than which conditions aid and hinder implementation.<br>e. Papers where investigation of the meaning or practice of PCC is neither presented in the aims or objectives of the study, nor in the results section of the paper, and any mention of PCC is merely made in the conclusion as a personal interpretation of the results by the author.<br>f. The concept of patient-centred care can be seen as including many subcategories such as patient-centred communication, patient-centred access, patient-centred outcomes, patient-centred diagnosis, shared decision-making, person-centred life-expectancy disclosure etc. Papers focusing exclusively on one deemed sub-component of PCC were not included as this would undermine the feasibility and specificity of this review. We wish to investigate PCC as a broad construct with wide applicability rather than specific constructs such as access and communication, though we recognise that work in these specific areas would be insightful.<br>g. Studies claiming to have performed empirical work to inform a ‘person-centred’ intervention that do not report the results of this work.<br>h. Studies in which the diagnostic group of focus for the majority of included participants is not a serious illness as defined and listed by Kelley et al.<br>i. Studies focusing on ‘personhood’. Personhood is a theoretical and philosophical construct concerned with the self that PCC draws on and is grounded in, rather than a model of care practice in itself.[2]<br>j. Studies focusing on ‘personalised medicine’ or ‘personalised gene therapy’.<br>k. Studies published in any language other than English.

Table 4 indicates the methods used to calculate quality scores for included studies using the Standard Quality Criteria developed by Kmet et al [3].

Table 4: Methods to calculate summary scores for Kmet et al. quality checklist

Summary score for quantitative studies	Summary score for qualitative study
**Total sum** = (number of “yes” * 2) + (number of “partials” * 1)<br>**Total possible sum** = 28 – (number of “N/A” * 2)<br>**Summary score:** total sum / total possible sum	**Total sum** = (number of “yes” * 2) + (number of “partials” * 1)<br>**Total possible sum** = 20<br>**Summary score:** total sum / total possible sum

2] Kitwood, T., *Dementia reconsidered: The person comes first*. Open University Press, Buckingham. 1997.

3] Kmet, L.M., R.C. Lee, and L. Cook, *Standard Quality Assessment Criteria for Evaluating Primary Research Papers from a Variety of Fields.* Conference proceedings. 2004.

Figure 1. A priori coding frame derived from Santana et al 2018 model of PCC

- S1. Creating a PCC culture
  - S1a. Core values and Philosophy of the organization
    - Addressing and incorporating diversity in care, health promotion and patient engagement
    - Patient and health-care provider rights
    - Patient-directed; integrating patient experience and expertise
    - Vision, Mission
  - S1b. Establishing operational definition of PCC
    - Common language around PCC
    - Consistent operational definitions
- S2. Co-designing the development and implementation of educational programs
  - Standardized PCC training in all health-care professional programs
    - Integration of all health-care sectors and professionals
    - Professional education and accrediting bodies
    - Translating into practice through continued professional education and mentorship
- S3. Co-designing the development and implementation of health promotion and prevention programs
  - S3a. Collaboration and empowerment of patients, communities and organizations in design of programs
    - Create patient advisory groups
    - Creating partnerships with community organizations
    - Identify resources
- S4. Supporting a workforce committed to PCC
  - S4a. Ensure resources for staff to practice PCC
    - Encourage teamwork and teambuilding
    - Provide adequate incentives in payment programs; celebrate small wins and victories
- S5. Providing a supportive and accommodating PCC environment
  - S5a. Designing health-care facilities and services promoting PCC
    - Areas or rooms that will support the accommodation of patients
    - Collaborate with and empower patients and staff in designing health-care facilities
    - Environments that are welcoming, comfortable and respectful
    - Facility that prioritize the safety and security of its patients and staff
    - Spaces that provide privacy
    - Spiritual and religious spaces
  - S5b. Integrating organization-wide services promoting PCC
    - Patient-directed visiting hours
    - Provide interpretation and language services
- S6. Developing and integrating structures to support health information technology
  - Common e-health platform for health information exchange across providers and patients
    - E-health adoption support through strategic funding and education
    - Electronic Health Record systems with capacity to coordinate and share healthcare interactions across the continuum of care
    - Health information privacy and security
- S7. Creating structures to measureand monitor PCC
  - Co-design and develop framework for measurement, monitoring and evaluation
    - Co-design and development of innovative programs to collect patients and caregiver experiences about care received and providing timely feedback to...
    - Reporting and feedback for accountability and to improve quality of health care

- P1. Cultivating communication
  - P1a. Listening to patients
    - Asking questions of what patients want to discuss (concerns, views, understanding)
    - Gathering information through active listening
    - Non-verbal behaviours (eye-contact, listening attentively, proximity or touch, head nodding)
  - P1b. Sharing information
    - Patients are provided with all the necessary information to make informed decisions in relation to their diagnosis and treatment plan
    - Sharing of information regarding patient's condition and their own impact or influences on their condition
  - P1c. Discussing care plans with patients
    - Acknowledging and discussing uncertainties
    - Aim and follow-up of treatment or interventions with possible outcomes and adverse events or side-effects
    - Creating a shared understanding
    - Discussing and building capacity of patients for self-management and self-care
    - Responding to patient and caregiver needs
- P2. Respectful and compassionate care
- P3. Engaging patients in managing their care
  - Co- designing care plans with patients
    - Care plans can be accessed by patients and health-care providers
    - Goal-setting
    - Shared decision making
    - Supporting self-care management
- P4. Integration of care
  - Communication and information sharing for coordination and continuity of care across the continuum of care
    - Between health-care providers
    - Discharge communication
    - Providing access to information and resources
    - Referrals to specialist

- O1. Access to care
  - O1a. Timely access to care
    - During consult, to be seen at emergency community care, pre-hospital, hospital, post-hospital; secondary care; time for patient care
    - Wait times for referrals to see specialists, to receive a consult
  - O1b. Care availability
    - Availability of health-care practitioners during and outside of working hours
  - O1c. Financial burden
    - Affordability of care including complimentary care and therapies, dental, pharmacare, ambulance
- O2.Patient-Reported Outcomes (PROs)
  - O2a. Patient-Reported Outcomes Measures (PROMs)
    - Functionality
    - Health-Related Quality of Life
    - Psychosocial outcomes
    - Symptoms
  - O2b. Patient-Reported Experiences (PREMs)
    - Assessment of care, including appropriateness and acceptability of care (competency, knowledge, skills of staff)
    - Recommendation or rating of hospital, health-care provider
  - O2c. Patient-Reported Adverse Outcomes (PRAOs)
    - Death
    - New or worsening symptoms
    - Unanticipated visits to health-care facilities

- O1. Access to care
  - O1a. Timely access to care
    - During consult, to be seen at emergency community care, pre-hospital, hospital, post-hospital; secondary care; time for patient care
    - Wait times for referrals to see specialists, to receive a consult
  - O1b. Care availability
    - Availability of health-care practitioners during and outside of working hours
  - O1c. Financial burden
    - Affordability of care including complimentary care and therapies, dental, pharmacare, ambulance
- O2.Patient-Reported Outcomes (PROs)
  - O2a. Patient-Reported Outcomes Measures (PROMs)
    - Functionality
    - Health-Related Quality of Life
    - Psychosocial outcomes
    - Symptoms
  - O2b. Patient-Reported Experiences (PREMs)
    - Assessment of care, including appropriateness and acceptability of care (competency, knowledge, skills of staff)
    - Recommendation or rating of hospital, health-care provider
  - O2c. Patient-Reported Adverse Outcomes (PRAOs)
    - Death
    - New or worsening symptoms
    - Unanticipated visits to health-care facilities
