# Supplementary material for: The empirical evidence underpinning the concept and practice of person-centred care for serious illness: a systematic review
Source: BMJ Glob Health. 2020 Dec 10;5(12):e003330. doi: 10.1136/bmjgh-2020-003330 (PMC7733074; doi:10.1136/bmjgh-2020-003330)
Supplement: online supplemental table 1 [file bmjgh-2020-003330supp002.pdf]

Supplementary Table 1. Data extraction table

*The specific aims/research questions relevant to this review are presented in bold text.

**Authors; Year of publication; Country**	**Aim and objectives***	**Study design, setting and methods**	**Sample**	**Main findings**	**Quality appraisal score (Kmet et al. scale [29]; possible range: 0-1)**
Bilodeau K., Dubois S. and Pepin J. (2014) [41]<br><br>Canada	Aim: To describe interprofessional patient-centred (IPPC) practice throughout the continuum of cancer care (diagnosis, treatment, recurrence and follow-up).<br><br>Research questions: **1) How do healthcare teams practice IPPC care at different stages of the cancer care continuum?** 2) What are the contextual factors influencing IPPC practice? and **3) What should IPPC practice consist of at different stages in the cancer care continuum?**	Qualitative multiple case study.<br>2 oncology interprofessional teams.<br>Interviews and observations.<br>Data analysed by content analysis.	Two oncology interprofessional teams.<br>Intentional sampling:<br>n=8 cancer patients (4 per team)<br>n=3 adult family members<br>n=20 health care professionals (nurses, doctors, physiotherapists, nutritionist and managers, and a psychologist, pharmacist, social worker and occupational therapist).	Three themes described current IPPC practice:<br>a) Welcoming the person as a unique individual, but still requiring the patient to comply<br>b) Paradoxical coexistence of patient-centred discourse and professional-centred practice;<br>c) Triggering team collaboration with culmination of patient's situation.<br><br>Three themes described IPPC practice participants desired:<br>a) Support in line with patient's experience and involvement<br>b) Respecting patients by not imposing professionals' values and goals<br>c) Consistency and regularity in collaboration of all members.<br><br>Patients stressed professionals' availability, sharing of information and professionals' attitude when describing IPPC practice.	0.7

Bisschop J.A.S., Kloosterman F.R., van Leijen-Zeelenberg J.E., Huismans G.W., Kremer B. and Kross K.W. (2017) [36]<br><br>The Nether-lands	Aim: **To investigate the experiences and preferences of head and neck oncology patients at the Oncology Center of MUMC** (in relations to the six dimensions of PCC as referred to by the American National Academy of Medicine).	Qualitative research design. Semi-structured interviews with patients.	n=19 patients<br>Patients were included if they had been treated at the MUMC Oncology Center for at least 6 months.<br>Patients who received palliative treatment were excluded.	Three dimensions of the IOM PCC definition predominated the interviews:<br>1) respect for patients' values, expressed preferences and needs<br>2) information, communication and education<br>3) involvement of family and friends<br><br>Within these dimensions, patients attached specific importance to three aspects:<br>a) provision of honest and complete information<br>b) an open discussion on decision-making with involvement of the patient<br>c) considering affection with family and friends as a crucial part in the treatment.<br><br>The dimensions of physical comfort, emotional support, relieving fear and anxiety and coordination and integration of care were of less significance according to the patients. However, comforting nervous patients was considered as crucial for a specialist in this field.<br><br>Within the coordination of care, remarkably low attention was given to waiting times on the day of appointment. In general, the coordination and planning covers more complex cases that need several appointments and patients expect the waiting times to be longer. The involvement of family and friends was deemed of great significance.	0.85

Calisi R., Boyko S., Vendette A. and Zagar A. (2016) [42]<br><br>Canada	Aim: **To investigate the understanding and practice of person-centred care by health care professionals and support staff at a cancer centre and to learn how patients and family members understand and experience person-centred care.**<br><br>**Research questions: 1) What does the phrase ''person-centred care'' mean to health care professionals and front-line staff working in the context of a busy cancer centre? 2) What does the phrase ''person-centred care'' mean to patients and family members? 3) What does person-centred care look like in practice for frontline and other staff and to patients and families?**	Sequential mixed methods approach involving 2 phases:<br><br>Phase 1 used large wall mounted posters and pens in public areas of the cancer centre to gather comments to answer the question ''What does person-centred care mean to you?''<br><br>Phase 2 used a 6-question, open-ended, paper-based questionnaire for staff and patients.<br><br>Manual coding technique was used to derive themes from both posters and questionnaires.	As posters were available in public areas the authors assume that staff, patients, family members, volunteers, and visitors had equal access to the posters, but it is not known who provided poster comments.<br><br>N=44 questionnaires were completed and returned:<br>n=30 front-line staff (n=19 radiation therapists; n=3 nurses; n=2 supportive care professionals; n=2 physicians; n=1 dental assistant n=1 genetic counsellor; n=1 student; n=1 clerk)<br>n=6 non front-line staff (n=2 technical staff, n=2 administrative assistant, n=1 coordinator, n=1 researcher)<br>n= 8 patients and patient and family advisors	97 themes were derived from posters and 134 themes derived from questionnaires. By combining common themes, it was concluded that staff, patients, and family members believe person-centred care to be:<br>1) care that is caring, compassionate, and empathetic<br>2) person or patient is the centre of focus<br>3) care is unique to the individual's needs<br>4) person or patient is a part of their care.<br><br>Furthermore, all staff, not only front-line staff, should provide person-centred care.	0.9
Chhatre S. , Gallo J.J., Wittink M., Sanford Schwartz J. and Jayadevappa R. (2017) [50]<br><br>USA	Aim: **To elicit patient stakeholders' experience and perspectives about patient-centred care.**	Qualitative. Discussion group.	n= 4 prostate cancer survivors<br><br>Patient 1 – age 71; underwent open radical prostatectomy with subsequent radiation and hormone therapy.<br>Patient 2 – age 59; received robotic radical prostatectomy.<br>Patient 3 – age 74; received proton therapy.<br>Patient 4 – age 65; retiree who received radical prostatectomy and subsequent radiation treatments.	The patients perceived patient-centredness to revolve around a theme of respect, engagement and dialogue between physician and patient; Instead of care being centred on one particular individual, be it patient or provider, it must be viewed as a collaborative process.<br><br>Patients reported wanting to be involved in treatment decisions, not simply told what to do. However, this does not imply that the physician and patient are equal in terms of health knowledge. They agreed that patient-centered care should not mean that patients can demand inappropriate treatments.<br><br>The degree of patient centredness was observed to be dependent on the situation; High severity conditions warranted a higher level of patient involvement, compared to mild conditions.	0.35

Colmer J and de Vries J. (2016) [53]<br><br>Ireland	Aim: To identify perspectives and experiences of care assistants with PCC in the nursing home in which they worked.<br><br>Objectives: **1) To address knowledge,** education and **attitudes around PCC** 2) To address obstacles and challenges around the implementation of PCC.	Semi-structured interviews revolving around 11 questions.<br><br>Setting: 2 nursing homes with a PCC policy (each with more than 100 residents, around 80% of whom had symptoms of dementia)<br><br>A phenomenological approach was used in the design of the interviews and the qualitative data analysis.<br><br>Participants' perspectives were extracted, emerging themes and sub-themes were identified. Data analysis utilised the Newall and Burnard (2006) method.	N= 13 care assistants<br><br>Inclusion criteria:<br>a) the carers had worked for at least one year in this role<br>b) they had been educated and received a diploma in 'Healthcare Support' which is a prerequisite to work as a care assistant in Ireland.	Findings showed considerable disparity between policy and practice, in particular because care assistants lacked clarity on what PCC is and reported that they were not educated in it. Among the 13 participants, 4 had not heard of PCC.<br><br>Nonetheless, carers' perspectives on 'good care' for people with dementia included elements of PCC which suggested its 'implicit' use in practice, such as respect, personal autonomy, privacy and dignity.	0.75
Cramm J.M., Leensvaart L., Berghout M. and van Exel J. (2015) [37]<br><br>The Nether-lands	Aim**: To explore views on what is considered important for Patient-Centred Care (PCC) among patients with end-stage renal disease and healthcare professionals in a haemodialysis department.**	Q methodology. Interviews were conducted asking participants to rank-order 35 statements representing 8 dimensions of PCC previously discussed in the literature. Participants explanations given during a follow-up interview, used to interpreted and verify the views found in the quantitative part of the analyses.<br><br>Views of PCC, and commonalities and differences between them, were explored using by-person factor analysis.	Purposive sampling, n=26 participants:<br>N=14 patients with end-stage renal disease receiving dialysis<br>N=12 healthcare professionals working at the haemodialysis department (n=2 doctors, n=6 nurses, n=4 staff members (i.e. 1 team leader, 1 policy advisor, 1 quality advisor, 1 social worker)).	Four views on what is important for PCC in end-stage renal disease were identified, suggesting that different patients may benefit from different types of care. These four views were:<br>1) listening to patients and taking account of their preferences in treatment decisions<br>2) providing comprehensible information and education to patients so that they can take charge of their own care<br>3) several aspects related to the atmosphere at the department<br>4) having a professional or acquaintance that acts as care coordinator, making treatment decisions with or for them.<br><br>All views agreed about the relative importance of certain PCC dimension: the patient preferences and information and education dimensions were generally considered most important, while the family and friends and the access to care dimensions were considered least important.	Qual: 0.9<br>Quant: 0.85

Edvardsson D., Fetherstonhaugh D. and Nay R. (2010) [44]<br><br>Australia	**Aim: To describe the content of person-centred care as described by people with dementia, family members and staff in residential aged care**	Qualitative explorative design using conversational interviews (individual, some by telephone) and focus groups.<br><br>Qualitative content analysis.	N=37 staff working in residential aged care facilities;<br>N=11 people with early onset dementia who had had experience of respite care;<br>N=7 home carers of people with dementia;<br>N=12 carers of people with dementia who lived in residential aged care facilities.	The findings indicated that the core category of person-centred care was promoting a continuation of self and normality.<br><br>Five content categories emerged as contributing to promoting a continuation of self and normality:<br>1) knowing the person<br>2) welcoming family<br>3) providing meaningful activities<br>4) being in a personalised environment<br>5) experiencing flexibility and continuity.	0.8
Galekop M., van Dijk H. M., van Exel J. and Cramm J. M. (2019) [40]<br><br>The Netherlands	**Aim: To explore professionals' and volunteers' views on PCC, and to see whether the views of the volunteers align with or differ from those of the professionals.**	Q methodology.<br>Interviews were conducted asking participants to rank-order 35 statements representing 8 dimensions of PCC previously discussed in the literature. Participants were also asked to elaborate on their ranking.<br><br>A by-person factor analysis was done to identify clusters in the ranking data. For each identified factor a weighted average ranking of the statements was computed, and interpreted and described as distinct views on PCC. Distinguishing statements and consensus statements were identified. Respondents' explanations of their ranking were used to verify the interpretations.<br><br>Possible differences in views between professionals and volunteers were inspected using the factor associations of respondents.	N=41 respondents:<br>n=30 professionals (21 nurses, 3 radiotherapists, 2 specialist geriatrics, 2 spiritual caregivers, 1 gastroenterologist and 1 palliative medicine doctor);<br>n=11 volunteers.<br><br>The participants were recruited from two hospitals and six hospices in the Netherlands.	The factor analysis revealed two distinct views on PCC, explaining 40% of the variance. Both viewpoints were supported by professionals and volunteers. The two main viewpoints identified were:<br><br>Viewpoint 1: 'The patient in the driver seat' – These respondents found it important that patients keep their autonomy during the last phase of life. According to them patients should always be in charge of their own care and professionals and volunteers should act according to the preferences of patients and should primarily support patients to achieve their goals.<br><br>Viewpoint 2: 'The patient in the passenger seat'. These respondents found PCC to be best delivered when professionals, volunteers and patients team-up and share control, with the patient in the passenger seat. In this view, whenever possible patients make their own choices, often after consultation with the professional. But when they are not willing or capable to decide themselves at any stage of their care, for example because they lack the energy or capacity to be involved, the professional should step in and decide on their behalf, in their interest.	Qual: 0.95<br>Quant: 0.85

Green M. , Anderson K., Griffiths K., Garvey G. and Cunningham J. (2018) [49]<br><br>Australia	**Aim: To a) identify the key components of patient experience that should be included in any experience of care measurements for Indigenous patients with cancer; and b) elicit participants' views on the appropriateness and likely acceptability of various data collection approaches for this patient group, from the perspectives of Indigenous people affected by cancer, and health professionals involved in care provision to Indigenous patients with cancer.**	Two rounds of semi-structured interviews and focus groups.<br><br>This study examined patient experiences as guided by the Picker Institute's Principles of PCC. Round One interviews were introduced with the definition of 'good quality cancer care' from the National Aboriginal and Torres Strait Islander Cancer Framework to highlight the person-centred orientation of the study team.	N=17 Indigenous people affected by cancer (either diagnosed with, or have cared for someone diagnosed with cancer)<br>N=28 health professionals (both Indigenous and non-Indigenous, whose work related to the care of Indigenous people diagnosed with cancer, including a broad range of clinical, supportive care, quality improvement and supervisory roles)<br>N=7 individuals in both aforementioned groups.<br><br>Recruitment occurred through a national web-based network and through four cancer services in urban and regional areas in three jurisdictions across Australia.	Several aspects of cancer care were identified as critical in shaping Indigenous patients' experiences. Key themes included:<br>a) feeling safe in the system<br>b) importance of Indigenous staff<br>c) barriers to care<br>d) the role of family and friends<br>e) effective communication and education<br>f) coordination of care and transition between services.	0.85
Kienle G.S., Mussler M., Fuchs D. and Kiene H. (2016 and 2018) (Two papers reporting data from one study) [51-52]<br><br>Germany	Aim of 2016 paper:<br>To investigate the concepts, therapeutic goals, procedures, and working conditions of integrative oncology doctors in the field of anthroposophic medicine.<br><br>Research questions**: 1) How are cancer patients cared for within the integrative care setting? 2) What are the underlying concepts and therapeutic goals? 3) What are the procedures? 4) How do expert physicians approach and assess cancer patients and which issues are important? 5) In what way is this treatment approach individualised and what does this mean? 6) What are the organisational working conditions?**	Qualitative study, using semi-structured, in-depth, individual interviews.<br><br>Data analysed using structured qualitative content analysis in combination with techniques from the thematic framework approach.	N=35 highly experienced integrative oncology doctors in the field of anthroposophic medicine, working in hospitals and office-based practices in Germany and other countries; sampled purposively.	2016 paper:<br>The emerging dimensions of the doctors' individualised approaches related to:<br>1) disease, condition, treatment focus<br>2) patient<br>3) doctor<br>4) therapy<br>Their treatments aimed at both tumour and symptom control and at strengthening the patient on different levels: living with the disease, overcoming the disease, enabling emotional and cognitive development, and addressing spiritual or transcendental issues according to the patient's wishes and anticancer and symptom-relieving treatments. Other external applications, nutrition and lifestyle advice, psychological support, and multiple forms of empowerment.<br>Their approach emphasised good patient-doctor relationships and sufficient time for patient encounters and decision-making. Individualisation appeared in several dimensions and was interwoven with standards and guidelines.<br>The doctors often worked in teams and cooperated with other cancer care–related specialists.	0.9

	Aim of 2018 paper:<br>To explore what role psychological, biographical, and spiritual factors play for experienced doctors working in integrative cancer care.<br><br>Research questions: 1) What roles do emotional, biographical, and spiritual issues play for physicians practicing integrative medicine? 2) What do physicians observe and experience with regard to these issues in the treatment process? **3) How do physicians support emotional and spiritual needs of patients? 4) What are the underlying treatment goals, concepts and themes?**			2018 paper:<br>Prevailing themes identified in this study were:<br>a) enabling patients to participate in life<br>b) promoting autonomy and coping<br>c) stabilising patients emotionally and cognitively<br>d) overcoming the disease<br>e) integrating spiritual issues.<br>Doctors offered conversation, counselling, and time, but also referred to art, music, literature, and nature, so that patients' ongoing emotional, psychological, and spiritual needs could be explored and addressed. Doctors' attitudes with regard to existential issues were seen as important, as was maintaining an attitude of openness towards existential issues.	0.9
Kvale K. and Bondevik M. (2008)[48]<br><br>Norway	Aim**: To get insight into patients with cancers' perceptions of the importance of being respected as partners and share control of decisions about interventions and management of their health problems and the reasons behind their wishes.**	Qualitative in-depth interviews. Interviews analysed according to Giorgi's step-by-step approach to phenomenology.	N=20 cancer inpatients, sampled purposively, with various cancer diagnoses at different stages and with different prognoses (n=10 women; n=10 men).	The units of meaning identified could be clustered into three themes with significance for patient centred care from patients' perspectives:<br>1) empowerment through being respected, listened to, believed, given honest information, and being valued;<br>2) shared decision making about the treatment of the disease (discussing treatment, but letting the doctor make the final decision)<br>3) partnership in nursing care, practiced by inviting patients to be partners in all decisions that affect their daily life and care, such as decisions about how to dress their wounds, administer chemotherapy and with whom they would share their rooms.	0.95

Nguyen T.K., Bauman G.S., Watling C.J. and Hahn K. (2017) [43]<br><br>Canada	Aim: To explore oncologists' perspectives on patient- and family-centered care (PFCC) to identify factors that influence their ability to practice PFCC.<br><br>Objectives**: 1) to explore oncologists' attitudes toward PFCC** and 2) to identify factors that influence their ability to practice PFCC	Exploratory, qualitative study. Individual semi-structured interviews. Two focus groups were then facilitated, consisting of previously interviewed participants to confirm and elaborate on the findings.<br><br>Thematic analysis was conducted, drawing on the principles of grounded theory.	N=18 oncologists (8 radiation, 4 medical, 4 surgical, 2 haematology-oncology).<br><br>Eligible participants were required to hold full-time staff positions practicing at least in part at the cancer institution. Trainees and general practitioners in oncology were excluded. Each focus group consisted of two radiation oncologists and one medical oncologist.	Three dominant themes emerged:<br>1) Physicians displayed cautious engagement in their approach to PFCC. Collectively, participants understood the general principles of PFCC. Physicians agreed that PFCC meant involving patients and families in care decisions and promoting patient autonomy. Other providers identified that their focus was ensuring patients had sufficient information and understanding for informed decisions. However, there was a limited understanding of the value, implications, and motivation for improving PFCC.<br>2) Both individual and system barriers to practicing PFCC were identified.<br>3) Physicians were able **to identify existing and potential PFCC behaviours that were feasible within existing system constraints**. These included improving physician-patient communication (e.g. by checking often for patient and family understanding, or consistently inquiring about the patient's financial situation, social history and support system), and streamlining care delivery (e.g. by having nursing staff provide additional teaching following the physician visit, or adequately informing patients of next steps and wait times between steps).	0.65

Oppert M.L., O'Keeffe V.J. and Duong D. (2018) [45]<br><br>Australia	Aim: to reveal the level of understanding that aged care workers have of person-centered care (PCC) principles, of the barriers that exist to prevent the practice of PCC and of the facilitators that promote it.<br><br>Research questions**: 1) What do aged care workers understand PCC to mean in the context of their workplace and role?** 2) What barriers do aged care workers believe exist to prevent the practice of PCC, and what facilitators do they believe exist to promote it?	Semi-structured interviews (containing 7 core questions), conducted in person or over the phone. Participants were also required to complete a questionnaire that sought demographic information such as age, qualifications and tenure as an aged care worker.<br><br>Data was analysed using thematic analysis. Furthermore, content analysis was used to quantify responses to enable comparative examination of aged care worker understanding of PCC across participants. The worker's understanding was classified according to how many factors of the VIPS framework (Brooker, 2007) were mentioned.	N=12 aged care workers (n=7 females, n=5 males) who provide direct care to aged residents.<br><br>All participants had a minimum qualification of Certificate III in Aged Care. Aged care workers who had a minimum 3-months experience in their role at this facility were eligible to participate.	Regarding research question number 1, findings revealed that aged care workers have a “reasonable but incomplete and superficial understanding” of PCC.<br>Of the sample, one participant embraced all 4 of the VIPS elements in their description (i.e. Valuing; Individualised approach; Perspective of service user; Social environment). Approximately three-quarters of the interviewed participants demonstrated a 'moderate to strong understanding' of PCC, in that they included two or three out of the four VIPS elements in their descriptions. A quarter of participants demonstrate 'superficial or limited knowledge' of PCC, in that they mentioned zero or one element of the VIPS framework. (Quotations included in results section illustrate examples of PCC understandings in more detail).	0.75
Ouwens M., Hermens R., Hulscher M., Vonk-Okhuijsen S., Tjan-Heijnen V., Termeer R., Marres H., Wollersheim H. and Grol R. (2009) [38]<br><br>The Netherlands	Aim: **to develop indicators of patient-centred cancer care and test them on a population of patients with non-small cell lung cancer, with the ultimate aim to improve present practice.**	Recommendations for patient-centred care were extracted from clinical guidelines and patients were then interviewed (semi-structured) to develop indicators for assessing the patient-centredness of cancer care.<br><br>These indicators were then tested with regard to psychometric characteristics, with data collected by means of questionnaires.	Interviews<br>N=30 head and neck cancer patients<br>N=7 patient representatives from the Dutch national association of patients with lung cancer<br><br>Questionnaire<br>N=132 patients with non-small cell lung cancer.	The authors developed 56 indicators for patient centredness covering the eight domains of PCC suggested by the Picker Institute. Interviewees found all the recommendations extracted from guidelines important, so all were included.<br>The criteria for waiting and throughput times came from the interviews as answers to the question of acceptable waiting times.<br><br>Furthermore, the patients added three specific information items:<br>a) information about the possible course of the disease<br>b) the possibility of a second opinion<br>c) information about the treatment option of “no active therapy”’.	Qual section score 0.65<br>Quant: 0.78

Pizzi M.A. (2015) [46]<br><br>USA	Aim: **to examine client-centered care at the end of life as that which enables engagement in meaningful occupation and promotes health and well-being until one dies.** (Part of a larger study of health professionals working in end-of-life care).	Open-ended semi-structured interviews conducted by telephone.<br>Thematic analysis.	N=3 occupational therapists<br><br>Inclusion criteria were being an active, working, end-of-life care occupational therapist for at least two years.	This paper reports the findings from one of the themes that emerged from the larger study: client-centered care.<br>Three sub-themes emerged within this theme:<br>1) Adaptation - all of the participants discussed how they adapt skills, routines, habits, or environments for people at the end of life, and how it can create a climate of trust, develop rapport, and establish well-being for people. They also discussed the importance of involving and supporting the family or significant others.<br>2) Client goals - participants expressed their client-centeredness through their descriptions of planning goals and meeting people's needs that were important from the perspective of the client and family<br>3) Choices - providing and facilitating engagement in daily choices was seen as an important form of empowerment for clients	0.65
Ross H., Tod A.M. and Clarke A. (2014) [47]<br><br>UK	Aim: to identify nurses' understanding of PCC and what factors facilitate such an approach to care within an acute medical ward.<br><br>Research questions**: 1) What do nurses understand by the term PCC? 2) How is PCC facilitated in the acute hospital medical ward?** 3) What are the implications for nurse education?	The study used an action research approach. Individual semi-structured interviews (and follow up group interviews or discussions).<br><br>Framework analysis was used to analyse the data.	Purposeful sample:<br>N=14 nurses (n=7 registered nurses; n=3 healthcare support workers; n=4 student nurses working on the ward)	Nurses had a 'clear understanding' of person-centred care in the context of their work. They discussed:<br>a) The importance of understanding more about the person and their personal identity, building relationships with the person, their family and within the care team;<br>b) Personal qualities, values and beliefs of staff that were congruent with PCC including listening to and recognising the importance of people's stories<br>c) Respecting the principles of person-centred care including recognising the importance of a person's wishes when considering care decisions and paying attention to all aspects of care that were important to the person.	0.8
Sjögren K., Lindkvist M., Per-Olof Sandman P-O., Zingmark K. and Edvardsson D. (2017) [54]<br><br>Sweden	**Aim: to explore factors characterising residential aged care units perceived as being highly person-centred, with a focus on organisational and environmental variables, as well as residents' and staff's characteristics.**	Cross sectional explorative design. Participating staff provided self-reported data and conducted proxy ratings on residents. Data were collected through a resident and a staff survey. The resident survey consisted of demographic variables, measurement scales on ADL-abilities and cognition. Person-centred Care Assessment Tool (P-CAT) was used to measure the extent to which staff perceive care provided as being person-centred.<br><br>Descriptive and comparative statistics, independent samples t-test, Chi2 test, Eta Squared and Phi coefficient were used to analyse data.	n=1460 residents and n=1213 staff data from 151 residential care units were collected, as well as data relating to characteristics of the organisation and environment, and data measuring degree of person-centred care.<br><br>The majority of participating care units were special care units for people with dementia (70%), located in rural (26%) and urban (74%) regions of Sweden. The unit size ranged from four to 26 residents, and between 4 and 20 permanent staff members. All residents living in the units were included in the study.	Highly person-centred residential aged care units were characterised by having:<br>a) a shared philosophy of care<br>b) satisfactory leadership<br>c) interdisciplinary collaboration and social support from colleagues and leaders<br>d) a dementia-friendly physical environment<br>e) staff having time to spend with residents<br>f) a smaller unit size<br>Residential aged care units with higher levels of person-centred care had a higher proportion of staff with continuing education in dementia care, and a higher proportion of staff receiving regular supervision, compared to units with lower levels of PCC.	0.91

Uphoff E. P. M. M., Wennekes L., Punt C. J. A., Grol R. P. T. M., Wollersheim, H. C. H., Hermens, R. P. M. G. and Ottevanger, P. B. (2012) [39]<br><br>The Netherlands	Aim: **to systematically develop evidence-based indicators, to be used to measure the quality of patient-centered cancer care as a first step toward improvement.**	RAND modified Delphi method.<br>First, key recommendations were identified from literature and were distributed over 5 domains of patient-centered cancer care: communication, physical support, psychosocial care, after-care, and organisation of care. These key recommendations were processed into a written questionnaire. A multidisciplinary panel of patients and medical professionals rated and prioritised these recommendations. Participants were asked ‘‘Please rate on a scale from 1-9 to what extent the execution of this action is a good measure for the quality of patient-centered cancer care.’’ To support their choice, panel members were provided with the source and evidence level of each key recommendation. Subsequently, the panel discussed the recommendations at a consensus meeting.	Multidisciplinary panel of n=14 patients and medical professionals<br><br>These consisted of: n=2 surgeons; n=2 medical oncologists; n=3 patients/patient representatives; n=1 radiotherapist; n=1 general practitioner; n=1 nurse-practitioner; n=1 nurse; n=1 psychologist; n=2 social workers.	Key recommendations were identified for communication (n=32), physical support (n=13), psychosocial care (n=25), after-care (n=11), and organisation of care (n=11). Merging all 92 key recommendations ultimately resulted in a core set of 17 quality indicators for patient-centered cancer care concerning criteria for communication skills, provision of information, examination of emotional health, appointment of a care coordinator, physical complaints, follow-up, rehabilitation, psychosocial effects of waiting times, and self-management.	Qual score 0.8<br>Quant score: 0.88
