# Supplementary material for: The empirical evidence underpinning the concept and practice of person-centred care for serious illness: a systematic review
Source: BMJ Glob Health. 2020 Dec 10;5(12):e003330. doi: 10.1136/bmjgh-2020-003330 (PMC7733074; doi:10.1136/bmjgh-2020-003330)
Supplement: online supplemental table 2 [file bmjgh-2020-003330supp003.pdf]

Supplementary Table 2. Retained studies' findings deductively mapped onto Santana et al. framework for person-centred care

Santana domains [34]	Number of supporting studies	Examples of corresponding codes from supporting studies
**S1. Creating a PCC culture**	5<br>[37,41-43,46]	*''Every employee is part of the patient-centred care approach and provides PCC. Sometimes, the focus is too much on frontline, and there is a lack of understanding other's people's roles.''* Researcher (Calisi, 2016, p313; Quality score 0.9)[42]
**S1a. Core values and Philosophy of the organisation**	2<br>[50,54]	*''The philosophy should pervade our whole institution. All should keep in mind the holistic nature of our interventions.'' Physician* (Calisi, 2016, p313; Quality score 0.9)[42]<br><br>*A shared philosophy of care (p <0.001, ŋ =0.090)* (Variable correlated to PCC from Sjogren, 2017, p4; Quality score 0.91)[54]
• Vision, Mission	0	No corresponding findings
• Patient-directed: integrating patient experience and expertise	1<br>[41]	*'The point was raised that patients should be invited to interprofessional meetings.'* (Bilodeau, 2015, p110; Quality score 0.7)[41]
• Addressing and incorporating diversity in care, health promotion and patient engagement	1<br>[49]	*All participant groups recognised that the presence or absence of an Indigenous care provider is a crucial aspect in shaping the experiences of cancer-affected participants, who described themselves as being "not as guarded" (302 CaAff) and feeling freer to ask questions without feeling silly, with an Indigenous person. As one participant recalled, "It was important to talk with an Aboriginal person – far more important than …the social worker for me."(303 CaAff)* (Green, 2018, p5; Quality Score 0.85)[49]
• Patient and healthcare provider rights	0	No corresponding findings
**S1b. Establishing operational definition of PCC**	1<br>[41]	*'This is why a number of professionals stressed that interprofessional collaboration should be improved in terms of the process, definitions and formalities.'* (Bilodeau, 2015, p110; Quality score 0.7)[41]
• Consistent operational definitions	0	No corresponding findings
• Common language around PCC	0	No corresponding findings
**S2. Co- designing the development and implementation of educational programs**	2<br>[47,54]	*"Students obviously don't know more than the (registered) nurses but sometimes they might have done assignments on different things. I've just done an assignment on dignity and privacy, so sometimes we can bring little bits of things (information) onto the ward...just reminding some staff and other students as well." Student nurse 3* (Ross, 2015, p1228, Quality score 0.8)[47]
**Standardised PCC training in all healthcare professional programs**	0	No corresponding findings

• Integration of all healthcare sectors and professionals	1<br>[42]	*''Holistic, multidisciplinary approach.'' Supportive Care Professional (Calisi, 2016, p31;* Quality score 0.9) [42]
• Professional education and accrediting bodies	0	No corresponding findings
• Translating into practice through continued professional education and mentorship	2<br>[39,51]	*'Regarding sources of knowledge, the doctors referred to colleagues in their direct working environment, to congresses and meetings (Onkofortbildung), including also discussions of experiences and presenting best and worst cases, to medical books and articles, case reports, guidelines, clinical trials and studies, to general conferences and, most important, to their experiences with patients and patient feedback.' (Kienle, 2016, p489;* Quality score 0.9)[51]<br><br>*A3. Communicative skills of all health care providers should regularly be evaluated, and feedback should be given.* (One of final set of approved quality indicators) (Uphoff, 2011, p35; Quality score 0.8 (qualitative), 0.88 (quantitative))[39]
**<u>S3. Co- designing the development and implementation of health promotion and prevention programs</u>**	0	No corresponding findings
**S3a. Collaboration and empowerment of patients, communities and organisations in design of programs**	0	No corresponding findings
• Identify resources	0	No corresponding findings
• Creating partnerships with community organisations	0	No corresponding findings
• Create patient advisory groups	0	No corresponding findings
**<u>S4. Supporting a workforce committed to PCC</u>**	3<br>[41,47,54]	'*In addition, the number of staff who received regular supervision was higher in units with high levels of PCC compared to in units with low levels of PCC (p =0.005, phi − 0.09)'* (Sjogren, 2017, p4, Quality score 0.91)[54]<br><br>*Flexibility in ward routines was spoken about as a facilitator to PCC; however, for this to occur, the leadership style had to be congruent with PCC: "If you've got a ward manager that is aware of people as individuals and encourages that, then you take your lead from them or your senior nurses...when you get new staff you should be encouraging them and setting a good example." (Research nurse 3)* (Ross, 2015, p1228; Quality score 0.8)[47]
**S4a. Ensure resources for staff to practice PCC**	0	No corresponding findings
• Provide adequate incentives in payment programs; celebrate small wins and victories	0	No corresponding findings

• Encourage teamwork and teambuilding	4<br><br>[37,41,46,51]	*"Professionals working as a team increase the right mood/atmosphere at the department, which also decreases the chance of making mistakes" (Patient 10)* (Cramm, 2015, p7; Quality score 0.9 (qualitative), 0.85 (quantitative))[37]<br><br>*"If the team is not ready to work as a team, throughout the process, it's not a question of asking me to do my job. It's a matter of being able to work together." (Professional 4)* (Bilodeau, 2015, p110; Quality score 0.7)[41]
**S5. Providing a supportive and accommodating PCC environment**	1<br><br>[49]	*Participants referred to the hospital environment and surroundings only in the context of cultural safety, including: the intimidating nature of the hospital environment; the presence or absence of Indigenous artwork and flags; the ability to engage in cultural practices, such as smoking ceremonies; space for multiple visitors in hospital (without judgment); and access to garden areas, enabling people to feel more relaxed, able to talk and to receive information.* (Green, 2018, p8; Quality Score 0.85)[49]
**S5a. Designing healthcare facilities and services promoting PCC**	3<br><br>[36,41,54]	*A dementia-friendly physical environment, ($p < 0.001$, $\eta = 0.045$)* (Variable correlated to PCC from Sjogren, 2017, p4; Quality score 0.91)[54]<br><br>*Team members thought proximity facilitated collaboration while physical distance between team members limited it: "I think that the distance between different members of the team can mean less consultation, so that if there were some problem, you deal with it much faster, instead of taking the time to call." (Professional 7)* (Bilodeau, 2015, p109; Quality score 0.7)[41]
• Collaborate with and empower patients and staff in designing healthcare facilities	0	No corresponding findings
• Environments that are welcoming, comfortable and respectful	5<br><br>[36,37,41,44,49]	*Linda, a family member narrated how a personalised environment supported seeing the person behind the disease: "In the facility, the residents are able to bring in their own things, like their photos and pieces of furniture and this makes all the difference. When you walk into the room, you know something about the person by what is there."* (Edvardsson, 2010, p2615, Quality score 0.8)[44]<br><br>*"It is important that the department is clean" (patient 5) (Cramm, 2015, p7*; Quality score 0.9 (qualitative), 0.85 (quantitative))[37]<br><br>*'Some respondents reported that their physical comfort in the waiting room is of minor importance in comparison to the reason why they were treated.' (Bishop, 2017, p2250;* Quality score 0.85)[36]<br><br>*The quality of the environment worried the professionals, who found the premises inappropriate for welcoming patients because they were small and did not foster a warm atmosphere. However, patients and families were much more concerned about the 'human environment' than the quality of the physical premises. (Bilodeau, 2015, p109;* Quality score 0.7)[41]
• Spaces that provide privacy	4<br><br>[36,39,47,48]	*It was important for the patients to be able to influence the decision about whom they had to share their room with, but for different reasons. Two reasons are illustrated in the following quotes: "I cannot stay in a dark room. I have to have the light on, night and day, and I have to have fresh air and an open door. I cannot be in the same room as patients who want to turn off the light and have the door closed. I told the nurses, and they tried to find patients who were willing to share the room with me. They never joked about it, and that was important because it is very serious for me. Sometimes the nurses put very*

		ill people in together with more healthy people in the same room. I have suffered much because of this. Lately the nurses have been cleverer by choosing patients that go together. They also ask me how I want things in my room." (Kvale, 2008, p586, Quality score 0.95) [48]<br><br>*A2. The health care provider should guarantee the following preconditions for a suitable conversation: a. providing a room with adequate privacy, with enough time for an accurate conversation.* (One of final set of approved quality indicators) (Uphoff, 2011, p35; Quality score 0.8 (qualitative), 0.88 (quantitative))[39]
• Spiritual and religious spaces	0	No corresponding findings
• Facility that prioritise the safety and security of its patients and staff	0	No corresponding findings
• Areas/rooms that will support the accommodation of patients	1<br><br>[49]	*Participants referred to the hospital environment and surroundings only in the context of cultural safety, including: the intimidating nature of the hospital environment; the presence or absence of Indigenous artwork and flags; the ability to engage in cultural practices, such as smoking ceremonies; space for multiple visitors in hospital (without judgment); and access to garden areas, enabling people to feel more relaxed, able to talk and to receive information.* (Green, 2018, p8; Quality Score 0.85)[49]
**S5b. Integrating organization-wide services promoting PCC**	1<br><br>[49]	*Health professionals also spoke about the benefits to staff of having an Aboriginal Liaison Officer (ALO) and/or Aboriginal Health Worker (AHW) in the service, including: helping staff understand why a patient/family member may be responding in a certain way; enabling the patient to trust enough to explain their concerns; and facilitating better linkages with services outside the hospital. "[O]nce they've seen that I've been able to work with the liaison officer, I've been able to build really strong relationships after that…..So it'shelped me to be introduced as a safe person..." (HP 205).* (Green, 2018, p8; Quality Score 0.85)[49]
• Provide interpretation and language services	2<br><br>[37,39]	*f. Adjusting information to the language skills of the patient and, when necessary, providing information in the native language of the patient* (One of final set of approved quality indicators) (Uphoff, 2011, p35; Quality score 0.8 (qualitative), 0.88 (quantitative))[39]
• Patient-directed visiting hours	0	No corresponding findings
**<u>S6. Developing and integrating structures to support health information technology</u>**	0	No corresponding findings
**Common e-health platform for health information exchange across providers and patients**	0	No corresponding findings
• Electronic Health Record systems with capacity to coordinate and share healthcare interactions across the continuum of care	0	No corresponding findings

• Health information privacy and security	0	No corresponding findings
• E-health adoption support through strategic funding and education	0	No corresponding findings
**<u>S7. Creating structures to measure and monitor PCC performance</u>**	0	No corresponding findings
**Co-design and develop framework for measurement, monitoring and evaluation**	0	No corresponding findings
• Co-design and development of innovative programs to collect patients and caregiver experiences about care received and providing timely feedback to improve the quality of health care (including complaints and compliments, wins and lessons learned)	1<br><br>[49]	*Most participants indicated that a face-to-face interview with a trusted person would be the best approach to measuring Indigenous patients' experiences of care. There was a clear preference among all groups for an opportunity for 'yarning', with several people suggesting a group or workshop setting.* (Green, 2018, p8; Quality Score 0.85)[49]
• Reporting and feedback for accountability and to improve quality of health care	1<br><br>[42]	*Accountability* (Arising theme from questionnaire; Frequency = 1) (Calisi, 2016, p312; Quality score 0.9) [42]
**<u>P1. Cultivating communication</u>**	11<br><br>[36,37,39,41-43, 47-51]	*Staff emphasized the importance of…communication between patient, family members and all members of the care team to provide individualized, compassionate care* (Calisi, 2016, p312, Quality score 0.9)[42]<br><br>*Per our patient stakeholders, two words characterise the roles of providers and patients – transparency and communication. Patients need to be honest and transparent and the providers need to make sure that patients can trust them. As in any relationship, without good communication, the relationship does not go far. (*Chhatre, 2017, p3; Quality score 0.35)[50]<br><br>*Attention to detail in phrasing – subtle changes can impact patient perception. (E.g. Using "What other questions do you have today?" in place of, "Do you have any more questions?")* (suggested component of *physician-patient communication) (Nguyen, 2017, Supplementary table 1;* Quality score 0.65)[43]
**P1a. Listening to patients**	9<br><br>[36-40,42,43,47,48]	*There was a clear recognition from participants that listening to and recognising the importance of people's stories were valuable in facilitating PCC* (Ross, 2015, p1228*;* Quality score 0.8)[47]<br><br>*"to be heard, acknowledged and taken care of.'' Patient* (Calisi, 2016, p131; Quality score 0.9)[42]<br><br>*Those holding [Viewpoint 2] indicated that according to their experience patients do want to tell their story several times, because it is part of their acceptation process and thus positively affects their well-being (11;− 3). "Them telling their story helps them understand what is going and gives some relieve, this is also sometimes the beginning of acceptance" (respondent 21).* (Galekop, 2019, p6; Quality score 0.95 (qualitative), 0.85 (quantitative))[40]

• Gathering information through active listening	1<br><br>[42]	*"Taking the time to ask questions and actually listening for their answers.'' Radiation Therapist* (Calisi, 2016, p312; Quality score 0.9)[42]
• Asking questions of what patients want to discuss (concerns, views, understanding)	4<br><br>[42,43,47,51]	*Ask for the patient and family's expectations for the current visit and work together to establish an agenda that addresses both patient and provider goals* (suggested component of *physician-patient communication) (Nguyen, 2017, Supplementary table 1;* Quality score 0.65)[43]
• Non-verbal behaviours (eye-contact, listening attentively, proximity/touch, head nodding)	3<br><br>[41,43,44]	*Patients also commented on professionals' empathy and attentiveness to them. A patient reported: "The attentiveness they give you. They take the time to speak to you. I often noticed that everyone looked you in the eye." (Patient 2) (Bilodeau, 2015, p110;* Quality score 0.7)[41]<br><br>*Pay close attention to the body language of patients and their caregivers and acknowledge these observations as appropriate*. (suggested component of *physician-patient communication) (Nguyen, 2017, Supplementary table 1;* Quality score 0.65)[43]
**P1b. Sharing information**	12<br><br>[36-42,44, 48,49,50,51]	*Patients attached specific importance to…provision of honest and complete information* (Bisschop, 2017, p2250*;* Quality score 0.85)[36]<br><br>"*Nothing about me without me.''* Patient (Calisi, 2016, p313; Quality score 0.9)[42]<br><br>*The need for effective communication and education was raised by both cancer-affected and health professional participants. Key points relating to information provision included: the importance of using accessible and appropriate language; using diagrams or drawings to aid comprehension; limiting the amount of information provided at any one time; considering the optimal timing of information provision; and recognising the need to repeat information over the course of the cancer journey.* (Green, 2018, p7; Quality Score 0.85)[49]
• Patients are provided with all the necessary information to make informed decisions in relation to their diagnosis and treatment plan	6<br><br>[37,38,42,43,48, 50]	*The patients added three specific information items, namely, 'information about the possible course of the disease', 'the possibility of a second opinion' and 'information about the treatment option of "no active therapy"'*. (Ouwens, 2010, p124; Quality score 0.65 (qualitative), 0.78 (quantitative))[38]<br><br>*The provider is responsible for explaining all treatment options and their ramifications, without being biased. (Chhatre, 2017, p3;* Quality score 0.35)[50]
• Sharing of information regarding patient's condition and their own impact/influences on their condition	3<br><br>[38,39,42]	*Additional suggestions from survey question 4 included…enabling patient access to their own test results…* (Calisi, 2016, p313*;* Quality score 0.9) [42]<br><br>*Patient knows which activities are allowed at home* (suggested patient-centred cancer care indicator) (Ouwens, 2010, p126; Quality score 0.65 (qualitative), 0.78 (quantitative))[38]
**P1c. Discussing care plans with patients**	8<br><br>[36,37,39,41-43,47,48]	*A patient with much pain expressed how important it was for medical staff to take time to discuss treatment with the patient: "The staff did not ask for my opinion the first six months. It was a long time before my pain was taken seriously. Why this was I don't know. Maybe I was not good enough at telling them, or I looked too healthy. The patient needs to be secure before talking about their needs. The nurses and doctors must have enough time to sit down and find out what the patient's*

		needs are. When the staff understood how bad it was, they took it seriously. Now I get things the way I want." (Kvale, 2008, p585; Quality score 0.95)[48]<br><br>*Participants also identified that involving patients and relatives in care decisions and care delivery in a compassionate manner supported PCC. (Ross, 2015, p1228;* Quality score 0.8)[47]
• Responding to patient and caregiver needs	11<br><br>[36-39,41,42,44,46,47,51,53]	*''PCC...you are looking after their needs opposed to just their physical needs. Like walking, breathing, eating, they need more than that'' (Care assistant 3)* (Colomer, 2016, p1162*;* Quality score 0.75)[53]<br><br>*Care is unique to the individual's needs (11.3%)* (Fifth most common theme arising from poster comments) (Calisi, 2016, p311; Quality score 0.9)[42]<br><br>*Christina, whose mother lived permanently in a residential aged care facility, described how family should feel welcome at all times: "The family should be told that 'this is your mother's home so come and go as you please".* (Edvardsson, 2010, p2615; Quality score 0.8)[44]
• Aim and follow-up of treatment or interventions with possible outcomes and adverse events/side-effects	4<br><br>[36,38,39,50]	*With the exception of two respondents, sufficient education was provided about the kinds of toxicities and expected adverse effects*. (Bisschop, 2017, p2248; Quality score 0.85)[36]<br><br>*"Specifically, I wanted information on 'after treatment, this is what you're going to face in life', and by 'this', I am speaking of the side effects that came with his particular treatment plan." (Patient stakeholder 2) (Chhatre, 2017, p3*; Quality score 0.35)[50]<br><br>*Specialists discussed aim and follow-up of the treatment with the patient.* (suggested patient-centred cancer care indicator) (Ouwens, 2010, p126; Quality score 0.65 (qualitative), 0.78 (quantitative))[38]
• Discussing and building capacity of patients for self-management and self-care	3<br><br>[38,39,46]	*Patient knows at discharge which medication to take and why.* (suggested patient-centred cancer care indicator) (Ouwens, 2010, p126; Quality score 0.65 (qualitative), 0.78 (quantitative))[38]<br><br>*E5. Depending on the individual patient, the health care provider should stimulate self-management and offer the proper information and support.* (One of final set of approved quality indicators) (Uphoff, 2011, p35; Quality score 0.8 (qualitative), 0.88 (quantitative))[39]
• Acknowledging and discussing uncertainties	1<br><br>[39]	*b. giving verbal information about the possible physical and psychosocial impact of diagnostics and the treatment on the patient* (One of final set of approved quality indicators) (Uphoff, 2011, p35; Quality score 0.8 (qualitative), 0.88 (quantitative))[39]
• Creating a shared understanding	4<br><br>[38,39,43,51]	*Check often for patient and family understanding.* (suggested component of physician-patient communication) (Nguyen, 2017, Supplementary table 1; Quality score 0.65)[43]<br><br>*Medical language had to be made understandable for patients: "Many patients do not understand the language of the doctors and many doctors do not understand the language of the patients…They have to…understand the technical terms. I tell them: translate it… This is very, very important. (General practitioner)* (Kienle, 2016, p488; Quality score 0.9)[51]

<u>**P2. Respectful and compassionate care**</u>	13<br><br>[36-38,40-42,44-48,50,51]	*The doctors also described working on their own attitudes toward the patient: having respect, and seeing the side of the patient's personality that impressed them, being dedicated to the care of very advanced and severely ill patients, and always looking for possibilities for providing support and relieving suffering.* (Kienle, 2016, p489; Quality score 0.9)[51]<br><br>*The interviewer then asked what the nurses said or did that gave them the feeling of being treated with respect. The following units of meaning were identified: "The nurses take me seriously and treat me as an adult and are very good listeners. They encourage me to tell them my wishes, listen to my questions and always give me an answer. They also showed me respect as an individual, not only as a patient, when they remembered my name without looking at my papers when I came back to the ward for treatment. The nurses respect me when doing something extra to help me, for instance finding something that is better for me to eat when I tell them that I cannot have the food they are giving me, rather than saying 'Oh, well...'"*. (Kvale, 2008, p585; Quality score 0.95)[48]<br><br>*"This professional, I took a dislike to him the day they told us he had [cancer]...He told us 'this is important bang bang'. It seems to me that he lacked compassion." (Family member 3)* (Bilodeau, 2015, p110; Quality score 0.7)[41]<br><br>*Professionals and volunteers consider dignity and respect and quality of life as the foundation of good care-delivery; "People are different, some even rude but they all deserve to be treated with dignity" (respondent 29). (Galekop, 2019, p6*; Quality score 0.95 (qualitative), 0.85 (quantitative))[40]
**P2a. Being responsive to preferences, needs and values**	16<br><br>[36-48,51-53]	*You've got to ensure that, not just the physical aspects of the care are taken, as you're doing your job, but that you're taking the person's wishes into consideration as well.'' (Care assistant 1)* (Colomer, 2016, p1162; Quality score 0.75)[53]<br><br>*Respondents with [Viewpoint 1] perspective believed that consideration of patients' preferences was an important aspect of PCC, as evidenced by the importance of treating patients with dignity and respect (item 1; staff member 4 and patient 11 stated that professionals should 'take the patient seriously and respect their choices'), improving quality of life (item 2), and involving patients in decisions about their care (item 4). (*Cramm, 2015, p5; Quality score 0.9 (qualitative), 0.85 (quantitative))[37]<br><br>*All participants described knowing the history, preferences, needs, interests and particularities of the person receiving care as being fundamental in the provision of person-centred care.* (Edvardsson, 2010, p2614; Quality score 0.8)[44]<br><br>*Respondents further explained that care should be provided keeping patients' preferences in mind (3;+ 2, 8;+ 2*): "I think the patient needs to express what he or she wants and the care will then be provided according to his or her needs" (respondent 5). (*Galekop, 2019, p4; Quality score 0.95 (qualitative), 0.85 (quantitative))[40]
• Acknowledge the patient as an expert in their own health and as a part of the healthcare team	7<br><br>[37-42,48]	*''I am the head of my health care team…ultimately how things progress in my care circle is my decision.'' Patient* (Calisi, 2016, p313; Quality score 0.9)[42]<br><br>*[Respondents with Viewpoint 2] especially felt that patients being in charge of their own care (item 28) was very important, as demonstrated by the following statements: "The patient has to make the final decision" (nurse 1); "The patient should be autonomous" (staff member 2); "Everything I can decide, I will decide" (patient 13)* (Cramm, 2015, p5; Quality score 0.9 (qualitative), 0.85 (quantitative))[37]

• Understanding patient within his/her unique psychosocial or cultural context (i.e: awareness of religious, spiritual, lifestyle, social and environmental factors)	13<br><br>[36,38,39,41-47,49,51,52]	*It referred to informing and shared decision-making, to addressing the mental and spiritual level ("What individualizes and forms a human being are our mental and spiritual forces . . ." [Gastroenterologist]) and to tailoring the whole treatment concept to the patient's condition, constitution, needs, and values: "There are many things that patients bring with them…what is their concept and to start from there, where the patient really stands…which ideas does the patient have about what is good for him/her and how can he/she build up his/her health further." (Internist)* (Kienle, 2016, p482; Quality score 0.9)[51]<br><br>*Consistently inquire about the patient's financial situation, social history and support system.*<br>(suggested component of physician-patient communication) (Nguyen, 2017, Supplementary table 1; Quality score 0.65)[43]<br><br>*Participating staff felt strongly that it was not enough just to know the individual; this knowledge had to be translated into practice and actively used in the provision of care so that it could be person-centred. Knowing the individual was described as essential for initiating conversations, activities and routines that were meaningful for the person. Also, staff could, with such knowledge, provide small extras that the person enjoyed such as sitting in the sun, going for a coffee and/or doing a bit of gardening.* (Edvardsson, 2010, p2614; Quality score 0.8)[44]<br><br>*The context in which cancer occurs is an important factor in shaping how cancer care is experienced. Key contextual factors identified by participants included past and present experiences of racism and discrimination, the underlying patterns of illness in the Indigenous population, health system characteristics and the varied life circumstances of patients. Many participants referred to the lack of open discussion about cancer ("the 'C' word"; 512 CaAff:) in the Indigenous community, for reasons including stigma, large amounts of existing stress, and different ways of dealing with challenges.* (Green, 2018, p5; Quality Score 0.85)[49]
• Responding empathically	2<br>[42,47]	*Care that is caring, compassionate, and empathetic (26.8%)* (Most common arising theme from poster comments; Frequency = 26) (Calisi, 2016, p311; Quality score 0.9)[42]
**P2b. Providing supportive care**	6<br><br>[38,41,45,47,52, 53],	*''It means give them a lot more time. Let them know that you are there for them. That would be my person centred care. To try make them happier.'' (Care assistant 10)* (Colomer, 2016, p1162; Quality score 0.75)[53]<br><br>*Theme 1: Support in line with patient's experience and involvement* (Bilodeau, 2015, p110; Quality score 0.7)[41]<br><br>*In practice, this might mean what this ACW understands PCC to be, "playing along with those dementia residents and understanding their perspective and adjusting your way in accordance with how they think about things", (ACW5, [Aged care worker 5]). In this instance, ACW5 is aware that the world of the person with dementia is different to that of his own and demonstrates an understanding of the unique perspective (P) of the person with dementia.* (Oppert, 2018, p686; Quality score 0.75)[45]
• Building a partnership with patients	11<br><br>[36,38,40-42,44,47-49,51,52]	*The experience of cancer was demanding for patients and they appreciated developing genuine relationships with the professionals*. (Bilodeau, 2015, p107; Quality score 0.7)[41]<br><br>*The essence that emerged was that the patients wanted to take part in all decisions about their daily life and care. This can be seen as a wish for partnership in nursing care. The following units of meanings were identified: "The nurses ask me to tell them my wishes and they do what I want. This is very good. I want to take part in decision making. When dressing my wound*

		with bandages and things like that, they ask if it is painful. If I had not agreed with the way the nurses are doing the dressing, they most certainly would have changed it." (Kvale,2008, p585; Quality score 0.95)[48]<br><br>*Health professionals talked about the importance of developing trusting relationships to overcome this: "I think probably the biggest thing is trust and in palliative care it frequently takes several visits to develop that trust. It's still really important with every individual that we really actively try and engage with the patient and their community." (407 HP) However, this trust could be fragile; if broken it could be critical in defining the person's experience of care: "And they said, "Oh, Aboriginal people don't burn when they have radiation". And that's an outright lie … Because I was burnt red raw from radiation." (503 CaAff).* (Green, 2018, p5; Quality Score 0.85)[49]
• Providing resources	0	NA
• Sensitivity to emotional/psychosocial needs	10<br><br>[36,38,39,41,42, 44-46,51,52]	*Thirteen respondents reported that emotional/psychological support was not necessary; all stated that the possibilities were explained by the doctors. Four respondents and one partner were given support by a social worker. The respondents and partners who received help from a social worker experienced this as a positive experience: "I have had four appointments with the social worker, and if I needed more it could be arranged. I could count on her, in the beginning I did not think I would need it, but at some point I thought well why not, it is being offered and I could use the help." (Respondent no. 5)* (Bisshop, 2017, p2250; Quality score 0.85)[36]<br><br>*Psychological care, such as talking to the patient, psychotherapy, and counselling, was a central part of treatment, particularly in cases of progressive disease: "I actually see my main focus primarily in talking with the patient…" (General practitioner).* (Kienle, 2016, p487; Quality score 0.9)[51]<br><br>*"Person-centred care is the person being the centre of the care. So spiritually, physically, mentally looked after as a whole. Making sure that person has holistic care" (Aged care worker 4).* (Oppert, 2018, p686; Quality score 0.75)[45]
**P3. Engaging patients in managing their care**	8<br><br>[37,39-42,44,50,51]	*Person or patient is a part of their care (12.4%)* (Fourth most common arising theme from poster comments and questionnaire; Frequency = 26) (Calisi, 2016, p312; Quality score 0.9)[42]<br><br>*Respondents with [viewpoint 1] felt that patients being in charge of their own care (item 28) and receiving support to achieve this goal (item 29) were less important….[Respondents with viewpoint 2] especially felt that patients being in charge of their own care (item 28) was very important, as demonstrated by the following statements: 'The patient has to make the final decision' (nurse 1); 'The patient should be autonomous' (staff member 2); 'Everything I can decide, I will decide' (patient 13)* (Cramm, 2015, p5; Quality score 0.9 (qualitative), 0.85 (quantitative))[37]<br><br>*Respondents holding [Viewpoint 1] strongly believe that patients should be in charge of their own care and that professionals and volunteers should primarily support patients to achieve their goals (28;+ 4* (statement 28; score + 4*), 29;+ 3*). Participants strongly feel healthcare professionals and volunteers should respect patients' autonomy; "Well, I believe that patient autonomy is a priority and we adjust the care we provide accordingly. This means that one can sympathize with others, that 'nothing is set' and everything is well communicated, and that the patient has sovereignty. Sovereignty… well... actually more like autonomy" (respondent 1)… Those holding [Viewpoint 2] considered it most important that patients, volunteers and professionals work together as a team with the patient in the passenger seat…Professionals and volunteers in this viewpoint in a way thus play a central role in the decision-making process in this viewpoint and according to them*

		patients are fine with that (28;-2, 29;-1); "In the last phase of life [being in charge of their own care] is not necessary anymore. I think patients are allowed to expect that everything is going alright" (respondent 29), "People who are in the last phase oftheir life often say: you can make the decisions, I'm tired of doing that. If you make good decisions, I can just 'be' ill. Spare me all the choices"(respondent 6). (Galekop, 2019, p5-6; Quality score 0.95 (qualitative), 0.85 (quantitative))[40]
**Co-designing care plans with patients**	11<br><br>[36,37,41-44,46-48,50,51]	*Patients differ in their willingness and ability to be involved, and thus the physicians have to help them reach the appropriate decision.* (Chhatre, 2017, p4; Quality score 0.35)[50]<br><br>*Another way that participants expressed their client-centeredness was through their descriptions of planning goals and meeting people's needs that were important from the perspective of the client and family.* (Pizzi, 2015, p445; Quality score 0.65)[46]<br><br>*The principles of providing PCC involved recognising the importance of a person's wishes when considering care decisions. Even when the patient him/herself was unable to make the decision, the nurses and multidisciplinary team worked with families to consider what the best interests of the person would be. This often involved supporting the person or their family to ask questions when unsure about treatment or care decisions: "Lots of the times we are advocates for them (patients)...when the doctor walks away we say "Are you alright with everything you have been told, do you want to ask any questions?" They sometimes say they don't understand what has been said...I think as long as the person has (mental) capacity, then I think it (their view) does get listened to. I think on this ward they are really good, they really try to meet people's needs and take into account their beliefs and what they want.' (Registered nurse 4)* (Ross, 2015, p1229; Quality score 0.8)[47]
• Shared decision making	12<br><br>[36-43,46,48,51,52]	*They were often seen as overwhelmed and under a lot of pressure with conventional treatments planned to start right away. Making time for therapeutic talk with respect to disease circumstances and treatment decisions, including detailed informing about how to understand the disease, stage, therapeutic options, and statistics as well as the emotional and existential dimensions, was thus a practical aspect that doctors emphasized as enhancing autonomy and informed choice-making. For me that [autonomy] is most essential, also in regard to decisions that have to be made right after surgery, namely, chemo-, radiation-, anti-hormone therapy etc … there the course is already set for non-autonomy. … I try to slow down and say: 'First of all, you have time.'… And secondly, I think it is a complete different situation whether they do radiation, because the doctor said so and they didn't think about it, or because they dealt with the subject and decided: yes, I want this. And to take the time … this is where I start working on the subject of autonomy. (Gynaecologist) (Kienle, 2018, p128*; Quality score 0.9)[52]<br><br>*Two of the participants suggested that in their client-centeredness they "manipulated" the situation to enable choices, but ones that further occupationally engaged their clients. One participant expressed that the development of the emotional climate or helping people at the end of life engage in full expression of themselves was client- and family-centered. (Pizzi, 2015, p446;* Quality score 0.65)[46]<br><br>*Some of the patients wanted to put all decisions in the hands of the doctors. The reason for this is illustrated by the following quote: "I do not wish to take part in decision making. The doctors know what they are doing and what they do is right. I have so much respect for their profession that I do not believe that my decision will be better than theirs. I trust them; at least I want to. I leave the decision in their hands. They have not really asked about my opinion, but I have no need of them doing so either." Only a couple of patients wanted to decide for themselves. This is illustrated by the following quotes. "Yes, yes of*

		course I want to decide about my treatment. I belong to the so-called difficult group of patients. There are three types of patients: the ones that have given up and say, 'OK, this has happened to me. It is fate'; the group that believes the doctor is still holy and knows everything and so follows his advice fully; and the difficult ones like me who are asking questions." (Kvale, 2008, p585; Quality score 0.95)[48]<br><br>*'For my questions to be answered and for all my options to be communicated to me. For my decisions to be respected.'' Patient* (Calisi, 2016, p313; Quality score 0.9)[42]
• Goal-setting	4<br><br>[40,46,51,52]	*Another way that [end of life healthcare professional] participants expressed their client-centeredness was through their descriptions of planning goals and meeting people's needs that were important from the perspective of the client and family. In the hospice, the patient and family must agree upon the treatment plan and goals. Setting realistic short- and long-term goals may vary greatly between clients depending on their prognosis. The dying trajectory is individual, and people can optimize function within the limits of the disease process.* (Pizzi, 2015, p446; Quality score 0.65)[46]<br><br>*Doctors encouraged patients to actively participate in their existing social network and work life but also to develop new goals and meaningful personal changes by reflecting on biographical aspects, such as dreams and wishes, both new and old, or by asking patients what they would like to experience if their life were to be condensed to only a few years. It also is an overall concept, because it is about the question of meaning, because it is about the question of coping. That is also one of my first questions: what is the main thing that you would do in the time left–no matter how long it is–so that I can tell them that it will be integrated into the treatment so that they do not say, 'Because of chemotherapy, I couldn't do such and such.' (Gastroenterologist)* (Kienle, 2018, p129; Quality score 0.9).[52]
• Supporting self-care management	3<br><br>[38,39,46]	*"You need to get people away from thinking that their wellness is going to come from someone doing something for them. But maybe their wellness is more based on being taught how to take care of themselves – self-management – on many levels." (Occupational therapist 1)* (Pizzi, 2015, p445; Quality score 0.65).[46]<br><br>*E5. Depending on the individual patient, the health care provider should stimulate self-management and offer the proper information and support.* (One of final set of approved quality indicators) (Uphoff, 2011, p35; Quality score 0.8 (qualitative), 0.88 (quantitative))[39]
• Care plans can be accessed by patients and healthcare providers	3<br><br>[40,42,43]	*Adequately informing patients of next steps and wait times between steps. (One of physician identified patient- and family-centred strategies pertaining to streamlining care delivery.)* (Nguyen, 2017, Supplementary table 2; Quality score 0.65)[43]
**<u>P4. Integration of care</u>**	5<br><br>[36,39,41,51,54]	*Interdisciplinary collaboration (p <0.001, η =0.041) …[was] rated significantly higher in units with higher PCC compared to units with lower PCC. (Sjogren, 2017, p4*; Quality score 0.91)[54]<br><br>*The complex organization of services could also affect the experience of care. Another family member summarized this: "The system [the oncology clinic] is so complicated that it's like swimming in molasses. (Family member 3)* (Bilodeau, 2015, p109; Quality score 0.7)[41]
**Communication and information sharing for coordination and continuity of care across the continuum of care**	10	*First, the communication with the administrative staff was described as respectful with a problem-solving approach with the patient being a central part. Second, internal communication between the HNO department and other medical specialists was generally experienced as being up to date: "All doctors were informed about my story, my operation and the whole*

	[36-38,40-42,44, 46,47,49]	*process. Three surgeons operated on me. There were many different specialists and for me it was one team. I cannot complain." (Respondent no. 1).* (Bisschop, 2017, p2248; Quality score 0.85)[36]<br><br>[Suggested patient-centred cancer care indicators]: *Specialists involved knew patient's history; Patient knew how to reach specialists; Patient knew about being discussed in a multidisciplinary team of specialists; Specialists involved took care of the coordination; Patient knew which specialist is his main contact person; Oncology nurse was present during bad news consultation; Existence of an oncology nurse was known by patient; Patient knew how to reach oncology nurse; Oncology nurse knew patient's history; Oncology nurses took care of the coordination; Patient knew how to reach paramedic professionals; Paramedic professionals involved knew patient's history; Paramedic professionals involved took care of the coordination* (Ouwens, 2010, p126-7; Quality score 0.65 (qualitative), 0.78 (quantitative))[38]
• Between healthcare providers	2<br><br>[38,40]	[As above] [Suggested patient-centred cancer care indicators]: *Specialists involved knew patient's history; Specialists involved took care of the coordination; Oncology nurse knew patient's history; Oncology nurses took care of the coordination; Paramedic professionals involved knew patient's history; Paramedic professionals involved took care of the coordination* (Ouwens, 2010, p126-7; Quality score 0.65 (qualitative), 0.78 (quantitative) [38]
• Referrals to specialist	2<br><br>[39,43]	*Utilizing multidisciplinary clinics to decrease wait times and patient anxiety between specialist referrals. (One of physician identified patient- and family-centred strategies pertaining to streamlining care delivery.) (Nguyen, 2017, Supplementary table 2; Quality score 0.65)[43]*
• Discharge communication	3<br><br>[38,39,49]	[Suggested patient-centred cancer care indicators relating to follow-up]: *Home care knows about the patient's situation; Patient knows which activities are allowed at home; Patient knows which side effects to be aware of; Patient knows when to contact the primary care doctor or specialist; Patient knows at discharge which medication to take and why; The primary care doctor knows about the patient's situation; Patient follow-up takes place on an agreed schedule; Specialist has enough time during consultations; Patient can reach the specialist between consultations* (Ouwens, 2010, p126; Quality score 0.65 (qualitative), 0.78 (quantitative).[38]<br><br>*"I found that I was let down with one of the main services that I really…depended on…(after) being discharged from hospital. I had a bad experience. I was left to fend for myself. I had to maintain my house ….which resulted (in) me getting infected and.. a lot of follow-up with doctors and medication, which could have been avoided.." (305 CaAff).* (Green, 2018, p6, Quality score 0.85)[49]
• Providing access to information and resources	1<br>[42]	"Additional suggestions from survey question 4 included allowing enough time for encounters, providing adequate education and resources…" (Calisi, 2016, p313; Quality score 0.9)[42]
**O1. Access to care**	7<br><br>[36,37,39,41-43,49]	*Furthermore, patients would appreciate having access to the services of the team throughout their entire experience. For example, one patient reported that he would have liked to have access to psychological services a year after his treatments finished because that was when he really felt the need.* (Bilodeau, 2015, p110; Quality score 0.7)[41]<br><br>*Care that is accessible* (Theme arising from questionnaire; Frequency = 5) (Calisi, 2016, p312; Quality score 0.9)[42]

		Among cancer-affected participants, key challenges, especially for those needing to travel for treatment, included: logistical difficulties and costs associated with transport, accommodation and food; separation from family and support networks during a very stressful time; and costs associated with bringing family support to patients. "Going away and being treated – that's the biggest thing because you know one of the most important things when you're not well is to have your home. I think it's better to be treated at home because you might not have the people around you as you're going through treatment" (304 CaAff). (Green, 2018, p6; Quality Score 0.85)[49]
**O1a. Timely access to care**	5<br><br>[36,38,41-43]	*…For care to be consistent and timely: ''For appointment times to be accurate; to get tests done in a timely manner; my opinion being respected; to be heard, acknowledged and taken care of.'' Patient (Calisi, 2016, p313;* Quality score 0.9)[42]<br><br>*Adequately informing patients of next steps and wait times between steps.* (One of physician identified patient- and family-centred strategies pertaining to streamlining care delivery.) (Nguyen, 2017, Supplementary table 2; Quality score 0.65)[43]
• Wait times for referrals to see specialists, to receive a consult	3<br><br>[38,39,43]	*E2. The maximal waiting time between the visit to the general practitioner and the first visit to the hospital should be 5 working days. E3. The maximal duration of the diagnostic process should be in accordance with the professional measures for the specific type of cancer.* (Two of final set of approved quality indicators) (Uphoff, 2011, p35; Quality score 0.8 (qualitative), 0.88 (quantitative))[39]<br><br>*Adding patient opinions had added value concerning criteria for waiting times and information supply. The guidelines often recommended speeding up the diagnosis and starting treatment as soon as possible. However, they provided hardly any concrete information about acceptable waiting times. The criteria for waiting and throughput times in our study came from the patient interviews as answers to the question of acceptable waiting times… [*Suggested patient-centred cancer care indicator]: *Waiting time first visit lung specialist <5 days* (Ouwens, 2010, p124-6; Quality score 0.65 (qualitative), 0.78 (quantitative)). [38]
• During consult, to be seen at emergency community care, pre-hospital, hospital, post-hospital; secondary care; time for patient care	4<br><br>[36,38,39,42]	*D1. The health care provider should guarantee sufficient time and attention to the patient during appointments after the primary treatment has finished.* (One of final set of approved quality indicators) (Uphoff, 2011, p35; Quality score 0.8 (qualitative), 0.88 (quantitative))[39]<br><br>*Within the coordination of care remarkably low attention was given to waiting times on the day of appointment. (Bisschop, 2017, p2250*; Quality score 0.85)[36]
**O1b. Care availability**	4<br><br>[44,47,51,54]	*Time [for staff] to spend with residents (p <0.001, η =0.108) …[was] rated significantly higher in units with higher PCC compared to units with lower PCC.* (Sjogren, 2017, p4; Quality score 0.91)[54]<br><br>*Family members and participants with dementia further described that to be person-centred, staff had to be available and present. This included taking time for a cup of tea or to chat with residents and families. Furthermore, all participants agreed that being with residents had to be prioritised ahead of the completion of tasks in order to promote person-centredness.* (Edvardsson, 2010, p2616; Quality score 0.8)[44]
• Availability of healthcare practitioners during and outside of working hours	4<br><br>[38,41,44,47]	*Patient can reach the specialist between consultations* (Suggested patient-centred cancer care indicator) (Ouwens, 2010, p126; Quality score 0.65 (qualitative), 0.78 (quantitative)).[38]

		Patients and families appreciated seeing the same professionals and having ready access to them...Furthermore, the difficulty of contacting some professionals made patients feel that they should only call in case of emergencies. One family member explained: "I am reluctant to call [the professional] …unless it's a major problem, but if it were for a trivial question ... [I wouldn't call]." (Family member 2) (Bilodeau, 2015, p109; Quality score 0.7)[41]
**O1c. Financial burden**	1<br><br>[49]	*"So if a patient needs six weeks of daily chemo and needs to stay there, that's a big financial burden, especially if they've living in the regions and they need to come into the city for appointments, petrol money – you know, that was a big issue too". (504 Both*). (Green, 2018, p6; Quality score 0.85)[49]
• Affordability of care including complimentary care and therapies, dental, pharmacare, ambulance	1<br><br>[43]	*Considering patient convenience and resource availability when ordering investigations*. (Physician identified patient- and family-centred strategy pertaining to streamlining care delivery.) (Nguyen, 2017, Supplementary table 2; Quality score 0.65)[43]
**<u>O2. Patient-Reported Outcomes (PROs)</u>**	2<br>[42,51]	*Accountability (Arising theme from questionnaire; Frequency = 1) (Calisi, 2016, p312; Quality score 0.9)[42]*
**O2a. Patient-Reported Outcomes Measures (PROMs)**	1<br><br>[51]	*Themes and goals centered on understanding "disease as a path/journey"; to "live as a human being" by participating in life despite the disease; to regain activity, control, and hope; to reduce depression and anxiety; to "choose a new life" by increasing autonomy, resilience, and courage; to reorient toward positive goals, not just "anticancer", to gain "emotional freedom from cancer"; to come to terms with past trauma; and to pursue self-development, lifestyle changes, creativity, and reflecting on relationships with the self and others. (Kienle, 2016, p488; Quality score 0.9)[51]*
• Health-Related Quality of Life	6<br><br>[37,40,41,46, 49,51]	*Treating patients with dignity and respect (item 1), quality of life (item 2), and patients' involvement in decision making (item 4) were also considered to be important aspects of PCC [within Viewpoint 2].* (Cramm, 2015, p5; Quality score 0.9 (qualitative), 0.85 (quantitative)).[37]
• Symptoms	6<br><br>[37,38,40,48,49, 51]	*Healthcare professionals pay attention to pain management.* (Statement was ranked as important by participants within viewpoints 1 and 2) (Cramm, 2015, p5; Quality score 0.9 (qualitative), 0.85 (quantitative)).[37]<br><br>*Previous cancer treatments, tumor response, and side effects were registered, along with the patient's well-being, symptoms, functional abilities, and other disease-related conditions. (Kienle, 2016, p482; Quality score 0.9)[51]*<br><br>*[Suggested patient-centred cancer care indicators]: Patient gets support to control physical complaints such as pain, suffocation, nausea, blood coughs, tingling, weight loss and insomnia* (Ouwens, 2010, p128; Quality score 0.65 (qualitative), 0.78 (quantitative)).[38]
• Functionality	1<br><br>[51]	*Other patients did not accept functional losses due to extensive resection (such as partial resection of the hard palate in an 88 year old man), or did not accept the complete loss of sexual function. (Kienle, 2016, p482; Quality score 0.9)[51]*
• Psychosocial outcomes	5<br><br>[37,49,51-53]	*If the patient could not be cured, and particularly if the disease was progressing rapidly, then along with the continuous endeavor to stabilize the tumor situation and control symptoms, the psychological and emotional issues became an increasing focus of treatment: "The primary goal surely is the tumor. But because I know that often it cannot be reached, the*

		secondary goal becomes the primary, very evidently. So that no matter how it grows or doesn't grow, one can acquire a positive attitude towards life." (General Practitioner [GP]) (Kienle, 2018, p128; Quality score 0.9)[52]<br><br>*''When a client is receiving 'good' care or person-centred care, there is a noticeable difference in the behaviour. Almost always the client is happier, calmer, more open with the carer, which makes the carers' job much easier.'' (Care assistant 1)* (Colomer, 2016, p1162; Quality score 0.75)[53]<br><br>*Healthcare professionals pay attention to patients' anxiety about their situation.* (Statement was ranked as important by participants within viewpoints 1, 2 and 4) (Cramm, 2015, p5; Quality score 0.9 (qualitative), 0.85 (quantitative)). [37]
**O2b. Patient-Reported Experiences (PREMs)**	0	No corresponding findings
• Recommendation or rating of hospital, healthcare provider	0	No corresponding findings
• Assessment of care, including appropriateness and acceptability of care (competency, knowledge, skills of staff)	3<br><br>[39,42,49]	*A3. Communicative skills of all health care providers should regularly be evaluated, and feedback should be given.* (One of final set of approved quality indicators) (Uphoff, 2011, p35; Quality score 0.8 (qualitative), 0.88 (quantitative))[39]
**O2c. Patient-Reported Adverse Outcomes (PRAOs)**	0	No corresponding findings
• New or worsening symptoms	0	No corresponding findings
• Unanticipated visits to healthcare facilities	0	No corresponding findings
• Death	2<br><br>[49,51]	For instance, *some patients were not afraid of death but rather of losing certain sensibilities or fine cognitive functions* (Kienle, 2016, p482; Quality score 0.9)[51]<br><br>*Being away from one's own Country or traditional lands, including the possibility of dying off Country, was also a particular source of distress for some participants. "[Aboriginal care provider] really understood where I was coming from being off Country. They understood my fears about being off Country and especially dying off Country – what would happen to my spirit, how would they treat my body and the aunties were able to explain the process of what happened if I did pass off Country and what would happen to my body." (303 CaAff).* (Green, 2018, p6; Quality score 0.85)[49]

**Inductively-identified themes additional to Santana model**		
Family and friend involvement and support<br>• Involving family/friends in information-sharing and decision-making<br>○ Respecting the opinions and worries of friends/family<br>○ Providing family/friends with opportunities to ask questions<br>• Addressing the needs of family/friends	11<br>[36-40,42,44,46,47,49,51]	*"Involving the family is a massive part of person-centred care, as their family know everything about them; they just know them inside out…" (Registered nurse 4).* (Ross, 2015, p1228; Quality score 0.8)[47]<br>*Welcoming family was described as developing and maintaining trust in that the staff would actively communicate changes and significant events to the family; so that they did not have to constantly seek out information by making phone calls or asking the staff when they visited. Welcoming family was also described by staff as creating opportunities for beneficial teamwork, so that the family's unique knowledge about the person with dementia could be incorporated into care plans.* (Edvardsson, 2010, p2614; Quality score 0.8)[44]<br>*Respondents in both views state that it is important to attend to the preferences of patients first, and to those of the family thereafter…'Of course there are some meetings involving the whole family, but ultimately, it is the patient who decides and not the family' (respondent 13). (*Galekop, 2019, p4; Quality score 0.95 (qualitative), 0.85 (quantitative))[40]<br>*The involvement and respect to the opinions and worries of friends and family was considered very important for the majority of patients.* (Bisschop, 2017, p2250; Quality score 0.85)[36]<br>*[Suggested patient-centred cancer care indicators]: Family and friends had opportunities to ask the specialists questions; Family and friends had opportunities to ask the nurses questions.* (Ouwens, 2010, p126; Quality score 0.65 (qualitative), 0.78 (quantitative)).[38]<br>*C2. The care provider should gather information on the psychosocial and emotional health status of family and friends of the patient and adequately refer to specialists, depending on the diagnosed problems.* (One of final set of approved quality indicators) (Uphoff, 2011, p35; Quality score 0.8 (qualitative), 0.88 (quantitative))[39]<br>*It was noted that carers undergo sustained periods of dealing with multiple stressors, combined with a lack of attention to their needs and their welfare and little-to-no follow-up, and that this contributed to a sense of being disregarded once the person being cared for had passed away or had reached a less acute stage. "…the carer is the one that carries the load. You know they're the ones that are looking after the sick person as well as trying to manage family." (103 Both).* (Green, 2018, p8; Quality score 0.85)[49]
Promoting continuation of normality and self-identity<br>• Support for participating in regular personal life activities	8<br>[37,38,44-46,49,51,52]	*Themes and goals centered on understanding "disease as a path/journey"; to "live as a human being" by participating in life despite the disease; to regain activity, control, and hope.* (Kienle 2016, p483, Quality score 0.9)[51]<br>*The overarching themes of doctors were to help patients live with the disease and find their own way through it; to encourage them to participate in life and regain autonomy and a sense of control and self-efficacy* (Kienle, 2018, p128; Quality score 0.9).[52]<br>*For instance, some patients were not afraid of death but rather of losing certain sensibilities or fine cognitive functions: "For instance a patient with advanced oesophagus carcinoma wished to write several publications; he had a spiritual orientation and rejected chemotherapy because he feared cognitive impairments and emotional constraints; with regular intralesional*

Providing meaningful activities for inpatients		*mistletoe extract injections the oesophageal stenosis reopened, the patient could eat and kept well for a substantial time with a good QoL and pursued his writing and publishing activities."* (Gastroenterologist) (Kienle 2016, p482, Quality score 0.9)[51]<br><br>*Individually targeted activities were described not only as providing a meaningful content to the day, but also as a means in reaffirming the residents as individual persons who were able to do the things they enjoyed. Family and staff further described that such activities preferably were adapted to the individual person's ability so that their self-esteem could be boosted by the successful completion of activities, rather than feeling defeated and demoralised by being expected to undertake something that was beyond their capability.* (Edvardsson, 2010, p2615; Quality score 0.8)[44]<br><br>*Two of the participants suggested that in their client-centeredness they "manipulated" the situation to enable choices, but ones that further occupationally engaged their clients. One participant expressed that the development of the emotional climate or helping people at the end of life engage in full expression of themselves was client- and family-centered.* (Pizzi, 2015, p446; Quality score 0.65).[46]
Structuring service organisation to enable continuity of care and patient navigation - Simplification of care pathways to ease patient navigation - Appointment system structured to allow patients to see same professionals over time - Structures enabling flexibility in service delivery and care practice. - Establishing cooperation pathways across specialisms and institutions	10<br><br>[36-38,41,43,44, 46,47,49,51]	*Utilizing multidisciplinary clinics to decrease wait times and patient anxiety between specialist referrals; Having nursing staff provide additional teaching following the physician visit*. (Physician identified patient- and family-centred strategy pertaining to streamlining care delivery.) (Nguyen, 2017, Supplementary table 2; Quality score 0.65)[43]<br><br>*The complex organization of services could also affect the experience of care. Another family member summarized this: "The system [the oncology clinic] is so complicated that it's like swimming in molasses. (Family member 3)* (Bilodeau, 2015, p109; Quality score 0.7)[41]<br><br>*The most prominent negative experiences noted were due to seeing different doctors at subsequent appointments: "We had an appointment with our doctor, but then we received a message that a new doctor was scheduled to help us that day. We really did not like that, especially because he had to tell us new test results and the prognosis. There was no explanation; they only told us our regular doctor was absent." (Respondent 12).* (Bisschop, 2017, p2250; Quality score 0.85)[36]<br><br>*Mostly in data from staff and family, it emanated that to be person-centred, aged care facilities need to have flexible routines adapted to the person with dementia's needs rather than the needs of staff, especially in relation to staffing, care tasks and activities* (Edvardsson, 2010, p2616; Quality score 0.8)[44]<br><br>*Some doctors worked in a cancer center…the others cooperated with oncologists, surgeons, radiotherapists, and other relevant specialists, often referring patients to each other. This cooperation was usually described as positive: "These centers know me all for long . . . they know that I know exactly what they do . . . it functions well" (Pediatrician).* (Kienle 2016, p488, Quality score 0.9)[51]
